# Trends of National and Subnational Incidence of Childhood Cancer Groups in Iran: 1990–2016

**DOI:** 10.3389/fonc.2019.01428

**Published:** 2020-01-14

**Authors:** Mahsima Shabani, Sahar Saeedi Moghaddam, Bahar Ataeinia, Nazila Rezaei, Farnam Mohebi, Bahram Mohajer, Kimiya Gohari, Ali Sheidaei, Farhad Pishgar, Moein Yoosefi, Farzad Kompani, Farshad Farzadfar

**Affiliations:** ^1^Non-Communicable Diseases Research Center, Endocrinology and Metabolism Population Sciences Institute, Tehran University of Medical Sciences, Tehran, Iran; ^2^International Hematology/Oncology of Pediatrics Experts, Universal Scientific Education and Research Network, Tehran, Iran; ^3^Department of Biostatistics, Faculty of Medical Sciences, Tarbiat Modares University, Tehran, Iran; ^4^Department of Epidemiology and Biostatistics, School of Public Health, Tehran University of Medical Sciences, Tehran, Iran; ^5^Division of Hematology and Oncology, Children's Medical Center, Pediatrics Center of Excellence, Tehran University of Medical Sciences, Tehran, Iran; ^6^Endocrinology and Metabolism Research Center, Endocrinology and Metabolism Clinical Sciences Institute, Tehran University of Medical Sciences, Tehran, Iran

**Keywords:** neoplasms, child, incidence, mortality, Iran

## Abstract

**Background:** Childhood cancer is a double-edged sword, considering its high rate of response to treatment despite a high vulnerability to develop future malignancies in survivors. Thus, multidisciplinary preventive, curative, and supportive strategies must be incorporated in childhood cancer care that require understanding the distribution and trend of cancer in the target population. In this article, we aimed to report the national and subnational trends of childhood cancer incidence in Iran from 1990 to 2016, and mortality/incidence ratio (MIR), which, to our knowledge, have not been reported in previous literature.

**Method:** Data on the incidence and mortality rates were collected from the National and Subnational Burden of Diseases project. We employed a two-stage spatiotemporal model to estimate cancer incidences by sex, age, province, and year based on the primary dataset of national death registration system. National and subnational age and gender-specific trends as well as MIR were calculated.

**Result:** The age-standardized incidence rate had a steady increasing trend for cancers in both female [annual percent change (APC), 1.6%] and male (APC, 2.1%) patients. Not only there was an increasing trend in most provinces but also there was a 40% divergence in age-standardized incidence rate at subnational levels. Leukemia, lymphoma, neoplasms of the central nervous system (CNS), digestive tract, endocrine gland, and urinary tract were the leading causes of cancer comprising more than half of all cancers. There was a remarkable general decrease in MIR by 75% as a proxy of care quality.

**Conclusion:** Regarding the increased trend of childhood cancer incidence, there is an essential need to address the etiologic factors and establish preventive plans for childhood cancers. Despite the favorable outcomes observed in cancer care, commensurate health resource allocation must be applied to diminish the subnational disparities.

## Introduction

Childhood cancers are unique in several aspects. First, cancer predisposing environmental factors are quite different in children and adults since children have a limited time of exposure and higher vulnerability to environmental factors that may influence their development, maturation, and behavior (1). Second, children have higher life expectancies, and the adverse effects of chronic illnesses may bring irrecoverable morbidities both physically and mentally (1). On the other hand, childhood cancers respond better to treatment. Furthermore, a cancer in children may be accompanied by malignancies either because of immunosuppression therapy or as a component of a neoplastic syndrome (2, 3). Moreover, since children are dependent on their adult caregivers, many symptoms may be mistaken or neglected that could result in delayed referral for diagnosis and treatment abandonment (1, 3–5). Finally, survivors of childhood cancers are predisposed to future socioeconomic difficulties, which are concerning the public health gravely (6, 7).

Cancer is the second leading cause of death due to non-communicable disease in children under 20 (8). According to the American Cancer Society, ~1 in 285 children under 20 are diagnosed with cancer (9). Although neoplasms are relatively rare in children in comparison to adults, they still account for 4.74 deaths per 100,000 children (10). Among the non-communicable diseases, neoplasms are the leading cause of death globally in children of 5–14 years old (8). Owing to the improved primary healthcare services, the proportion of death attributed to cancers in comparison to communicable disease and injuries is rising especially in high and high-middle socio-demographic index countries (10).

According to sustainable development goals, a one-third decline in premature death due to non-communicable diseases and decreased under-5 mortality to at least 25 deaths per 1,000 live births are among targets to be reached by 2030 (11). Even though Iran has achieved the latter by 2015 (12), given the rising mortality of cancers and the better response of children to the cancer treatment, initiatives in directing health policies to childhood cancer prevention and treatment is essential for accomplishing both goals. To make effective health policies, robust documents in population-based cancer registries are required. Although several studies focused on regional cancer registries in Iran (13–15), there is a remarkable paucity of literature on national and subnational population-based cancer incidence and cancer trend reports in Iran.

Evaluating the cancer incidence rates and the trend of all-cause and cause-specific incidences in national and subnational levels are first-line studies in designing specific programs for the disease burden control. Therefore, we aimed to provide population-based reports of childhood cancer incidence rate, age- and gender-specific incidence distribution, and leading cancer subtypes along with a 27-year cancer incidence trend from 1990 to 2016. The quality of care is further evaluated by calculating mortality/incidence ratio (MIR) as an indicator of cancer management outcome. In this method, a decline in the ratio of crude rate of mortality to the crude rate of incidence indicates a better efficacy of cancer control program.

## Methods

### Data Source

Data were collected from the Iranian national program for cancer registry through the Cancer project. The Ministry of Health and Medical Education has been responsible for the management of cancer registries since 2000. Data for years before 2000 were extrapolated using the random intercept mixed effects model and the age-spatiotemporal model. The main sources for data collection included pathology, radiotherapy, chemotherapy, and surgical centers, hospitals, as well as death registries. After removing duplicate data, a unique dataset is developed. Main variables in this study included age, gender, and place of residence (at district level), codes of diagnosed cancers, year of initial diagnosis of cancer, first names, surnames, name of parents, postal address, and other identification characteristics. The same steps were performed for both children and adult patients.

The incompleteness of the cancer registry was addressed through comparison with the Social Security Insurance (SSI) organization registry. SSI covers almost 40% of population in Iran and has a complete registry of the financial insurance services of the registered patients with cancer. Given the extremely high cost of cancer medication and hospitalization, insurance organizations have an almost 100% coverage for the registered patients with cancer. We observed a 78% incompleteness in 2000 and a 25% incompleteness in 2010, based on SSI registry. Given the assumption that the cancer registry has worked similarly for other insurance organizations, we have used the same completeness rates for all cancer patients.

Population size and status, additional variables on wealth index, education years, and rate of urbanization were extracted from the Household Income and Expenditure Surveys, as well as Population and Housing Censuses of the Statistical Center of Iran (16). Except for the calculation of MIR, all the reported values are extracted from the Cancer subdivision of the National and Subnational Burden of Diseases project. We used the cancer mortality rates available in National and Subnational Burden of Diseases project from 1990 to 2015 and a recent report of childhood cancer mortality from our center, for calculating MIR (17–22).

### Definition

Classification of cancer types was defined by the International Classification of Diseases for Oncology, International Statistical Classification of Diseases, and Related Health Problems 10th Revision. Generally, we have included 18 main groups and 70 subtypes of cancer. The diagnosis of cancer was recorded by the associated physicians who initially examined the patients.

### Statistical Analysis

Statistical analysis was conducted in R programming platform and STATA software (version 11.0). Missing data were initially elicited from available individual data. Remaining missing data were computed through Amelia package in R programming language (23). Investigation for duplicated data was done by text-mining algorithm of Python software (version 2.7.4). All-cause cancer incidence rate was initially modeled and extrapolated by a random intercept mixed effect model, and then, residuals were remodeled by an “age-spatiotemporal” model, in which remaining observations were predicted by other observations based on defined weights (24). In case of location, weights were applied considering the neighborhood provinces, and in case of age groups, the weight matrix was an applied function of age difference among age groups combined with a smoothing hyperparameter. Data on incidence of cancer subtypes were modeled by multinomial logistic regression approach. We further applied a Bayesian model for multinomial distribution and overdispersion in this logistic regression. The uncertainty intervals of 95% were estimated through simulation of the aggregated data based on variations in individual data.

Decomposition analysis was done to find the contribution of population growth, population aging, and change in the age-specific incidence rate on the absolute change of cancer new cases. In this process, the age and sex structure and age-specific rates from 1990 were applied to the total population of the year 2016, which was called first hypothetical data. The difference between new cases in 1990 and the first hypothetical data were attributed to the population growth. Applying age-specific rates from 1990 to age and sex structure and population in 2016 was called second hypothetical data. Differences between the second and first hypothetical data were attributed to population aging. Attribution to change in the age-specific incidence rates was considered as difference between new cases in 2016 and the second hypothetical data (25).

Exponential regression coefficient from regression of natural logarithm (ln) of incidence rate is considered as annual percent change (APC) (26). Age-standardized rates were computed by direct method of standardization using the Iranian population in year 2016 and package “epitools” in R 3.0.2 (27).

## Results

**Figure 1:**
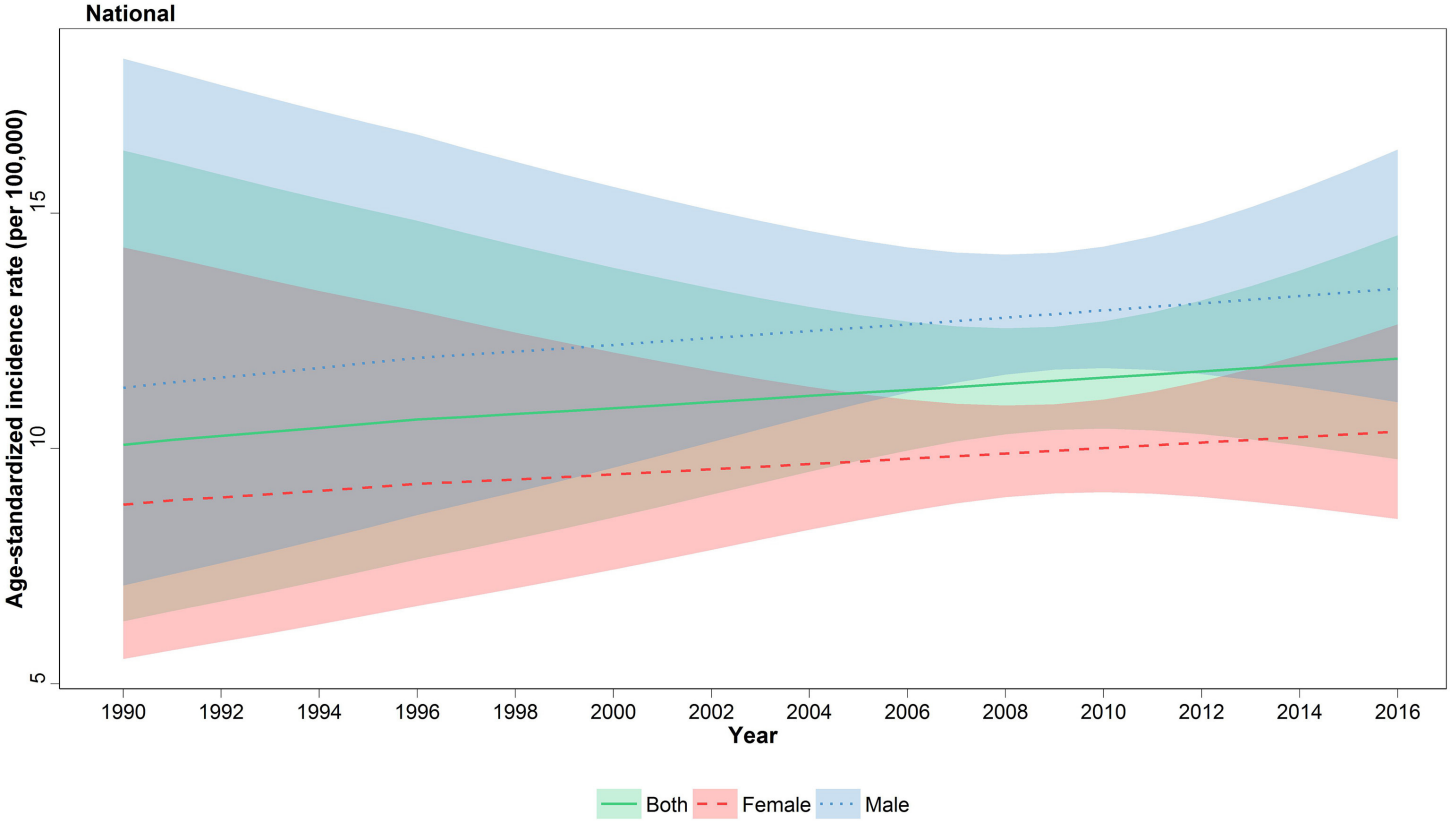
National incidence of childhood cancers, 1990–2016. Age-standardized incidence rate (ASR) of childhood cancers with associated 95% uncertainty intervals are illustrated for females, males, and both genders.

The age-standardized incidence rate (ASR) of all-cause cancers in children under 15 was 11.9 (95% uncertainty interval, 9.8–14.5) in 2016 with a 13% increase compared to 1990 with 10.08 (6.33–16.33) new cases per 100,000, at national level (Figure 1). On the other hand, total new cases of cancer patients have undergone a descending trend regarding 2,086 (1,712–2546) new cases in 2016 vs. 2,478 (1,556–4,014) in 1990; the calculated APC was positive for incidence rate (+0.57%) and negative for total new cases (−0.66%) during 27 years of study (Table 1). This ascending pattern in incidence rate was observed in both male (11.29, 7.09–18.28 in 1990; 13.4, 11–16.4 in 2016) and female patients (8.81, 5.53–14.27 in 1990; 10.4, 8.5–12.6 in 2016) (Figure 1, Table 1). The male/female ratio 1.3:1 in ASR remained constant during 27 years of study. To identify the effect of population growth on the observed variations in ASR, we conducted a decomposition analysis. There was a −27.3% change attributable to population growth, −1.4% change in age structure, and +12.9% change in incidence rate.

**Table 1:** National incidence of all-cause cancers in 1990 and 2016, by age groups and genders.

**Measure**		**New cases**			**Incidence rate (per 100,000)**		
**Age**	**Sex**	**Year**		**APC (%)**	**Year**		**APC (%)**
		**1990**	**2016**		**1990**	**2016**	
Under 15	Both	2,478 (1,556–4,014)	2,086 (1,712–2,546)	−0.66	10.1 (6.3–16.3)	11.9 (9.8–14.5)	0.64
	Female	1,055 (662–1,709)	889 (730–1,084)	−0.65	8.8 (5.5–14.3)	10.4 (8.5–12.6)	0.63
	Male	1,424 (894–2,306)	1,197 (982–1,462)	−0.66	11.3 (7.1–18.3)	13.4 (11–16.4)	0.66
Under 1	Both	112 (70–182)	117 (96–144)	0.17	7.4 (4.7–12)	9 (7.3–11)	0.73
	Female	48 (30–77)	50 (41–61)	0.17	6.5 (4.1–10.5)	7.8 (6.4–9.5)	0.72
	Male	64 (40–104)	67 (55–83)	0.18	8.3 (5.2–13.5)	10.1 (8.2–12.3)	0.74
1–4	Both	541 (340–877)	556 (455–680)	0.1	8 (5–13)	9.8 (8–11.9)	0.77
	Female	231 (145–374)	236 (193–288)	0.08	7 (4.4–11.3)	8.5 (7–10.4)	0.77
	Male	310 (195–503)	320 (262–392)	0.12	9 (5.7–14.6)	11 (9–13.4)	0.77
5–9	Both	929 (584–1,504)	698 (572–852)	−1.1	10.7 (6.7–17.4)	12.2 (10–14.9)	0.49
	Female	396 (249–641)	295 (242–360)	−1.12	9.3 (5.9–15.1)	10.6 (8.7–12.9)	0.48
	Male	533 (335–863)	402 (330–492)	−1.08	12 (7.6–19.5)	13.7 (11.2–16.7)	0.49
10–14	Both	896 (562–1,451)	715 (589–871)	−0.86	12.5 (7.9–20.3)	15 (12.3–18.2)	0.69
	Female	380 (238–615)	308 (253–375)	−0.8	11 (6.9–17.8)	13 (10.7–15.8)	0.65
	Male	516 (324–835)	407 (335–496)	−0.9	13.9 (8.8–22.6)	16.9 (13.9–20.5)	0.73

*Data in parenthesis are 95% uncertainty interval (UI)*.

*APC, annual percent change*.

**Figure 2:**
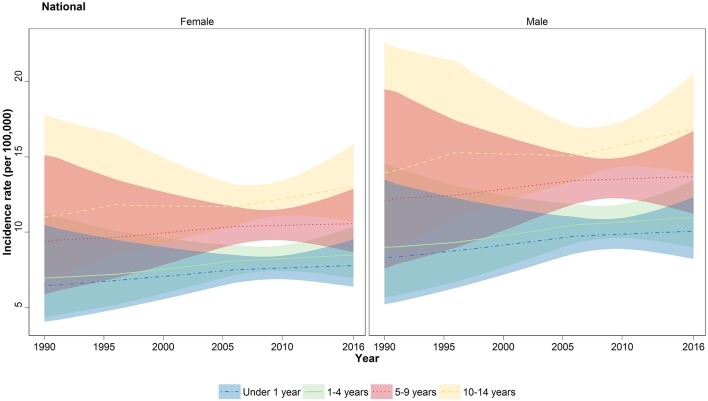
National trend of childhood cancer incidence by four major age groups. Incidence rate of all-cause cancers depicted in lines for under 1 year (blue), 1–4 years (green), 5–9 years (red), and 10–14 years (yellow). The colored areas shows the 95% uncertainty interval for the associated lines.

When analyzed in age subgroups, a similar incremental growth of incidence rate was observed in under 1, 1–4, 5–9, and 10–14 years of age subgroups (Figure 2). The highest APC among the age subgroups was observed in under 1 and 1- to 4-year-old children with values of 0.73 and 0.77%, respectively (Table 1). Moreover, within all years of under study, the incidence rate was raised by increasing the order of the age groups (Figure 2). The children of 10–14 years old had the worst rate in 2016 with 15 (12.3–18.2) new cases per 100,000, ~40% more than the average rate for all age groups except children of 10–14 years old (Table S1). A persistent male predominance was evident in all children age groups, and interestingly, the annual male/female ratio (1.3:1) did not undergo a significant change in all age groups during study period (Table S1).

At national level, the six leading causes of cancer in children under 15, which together accounted for ~80% of total cancer incidence in 2016, were leukemia, lymphoma, eye, brain, and other central nervous system (CNS) neoplasms, malignant neoplasms of thyroid and other endocrine glands (especially adrenal gland neoplasms), malignant neoplasms of digestive organs (especially those of liver origin), and urinary tract (especially neoplasms related to kidneys) (Table 2). However, in female gender, malignant neoplasms of female genital organs accounted for more than 10% of total cancer incidence rate, individually. One might bear in mind that this class of cancers is comprised of germ cell tumors, rhabdomyosarcomas, as well as other cancers of the external genitalia, ovary, vagina, and uterus origin.

**Table 2:** Gender-specific distribution of cancer subtypes in 1990 and 2016 and associated APC.

**Cause**	**Sex**	**ASR (per 100,000)**		**APC (%)**
		**1990**	**2016**	
Leukemia	Both	1.35	4.82	5.01
	Female	0.97	3.71	5.31
	Male	1.72	5.88	4.84
Central nervous system	Both	0.22	2.48	9.71
	Female	0.17	2	9.93
	Male	0.27	2.93	9.58
Lymphoma	Both	0.94	0.87	−0.30
	Female	0.71	0.66	−0.32
	Male	1.15	1.07	−0.29
Endocrine glands	Both	0.16	0.87	6.69
	Female	0.24	1.28	6.65
	Male	0.09	0.48	6.74
Digestive organs	Both	0.60	0.68	0.49
	Female	0.53	0.6	0.52
	Male	0.68	0.76	0.47
Urinary tract	Both	0.41	0.44	0.26
	Female	0.19	0.2	0.18
	Male	0.61	0.66	0.29
Other neoplasms	Both	6.48	2	−4.42
	Female	6.06	2.10	−3.99
	Male	6.87	1.91	−4.81

**Figure 3:**
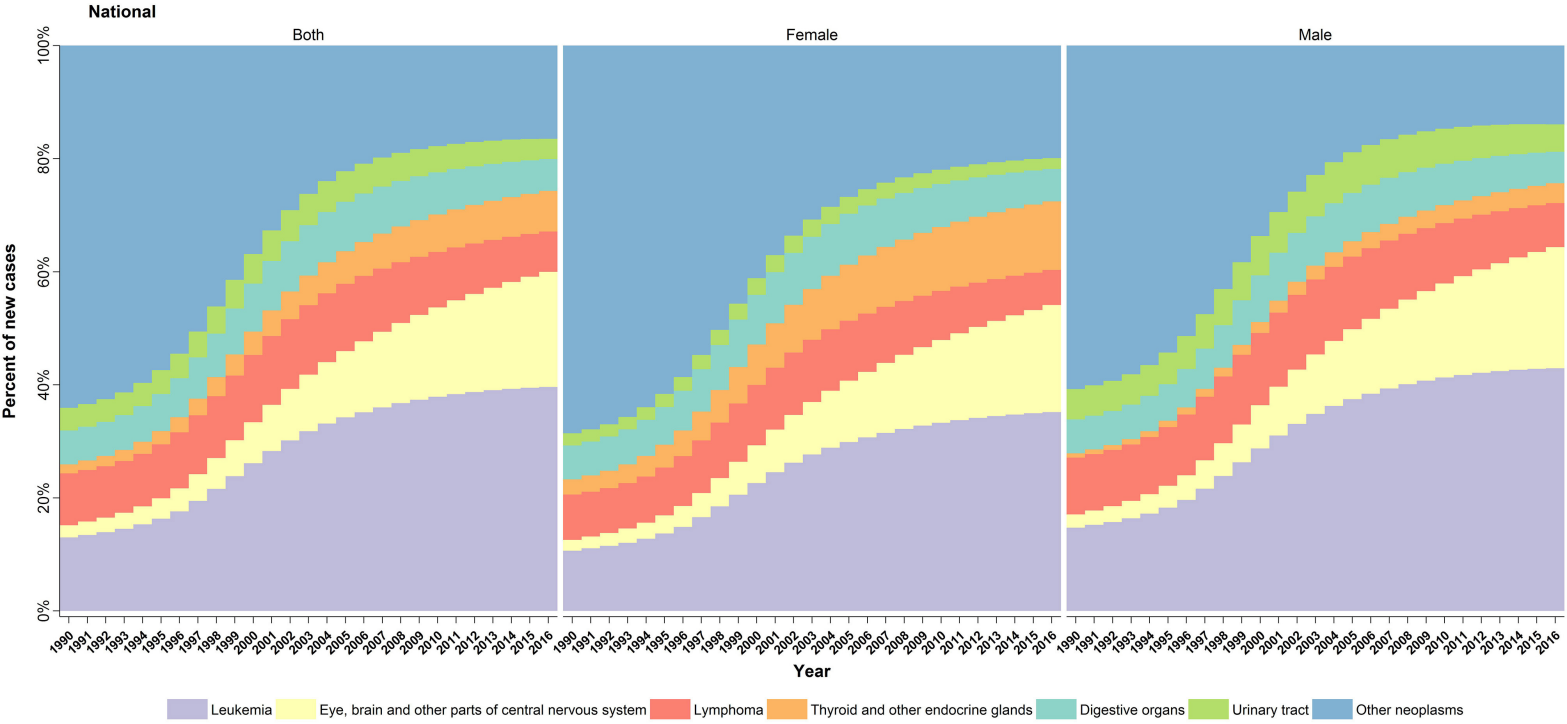
Cause-specific trend of childhood cancers incidence in Iran, 1990–2016. Annual changes in the percentage of new childhood cancer cases per six leading subtypes during the study period.

Notably, over the study period, similar to the total cancer incidence, all of the aforementioned cancer subtypes underwent an increasing trend from 5% (in urinary tract neoplasms) to 11-fold (eye, brain, and other CNS neoplasm), except for the lymphomas, which showed a decreasing pattern (Figure 3). Prominently, the male predominance was observed in all leading causes of cancer except for malignant neoplasms of thyroid and other endocrine glands group, in which a relatively constant male/female ratio of about 1:3 was recorded (Table 2).

**Figure 4:**
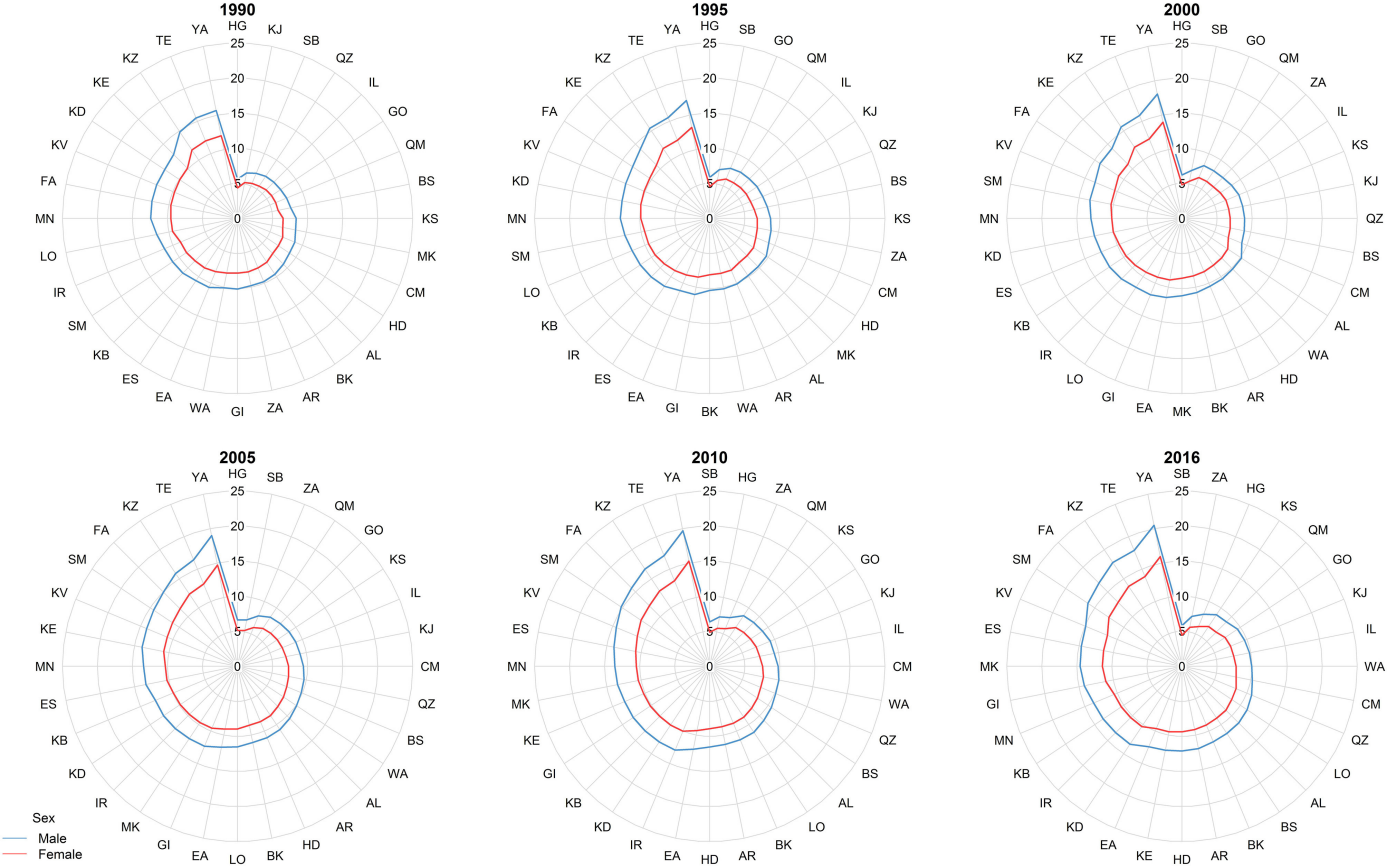
The subnational incidence rate of childhood cancers by gender in 1990, 1995, 2000, 2005, 2010, and 2016. The province-specific age-standardized incidence rates (ASRs) depicted in radar graphs to demonstrate the geographic and gender disparities. The two-digit codes mentioned in this figure are Hierarchical Administrative Subdivision Codes (HASC) of Iranian provinces from which “IR” has been eliminated to avoid repetition. IR, National; AL, Alborz; AR, Ardebil; BK, Kermanshah; BS, Bushehr; CM, Chahar Mahall and Bakhtiari; EA, East Azarbaijan; ES, Esfahan; FA, Fars; GI, Gilan; GO, Golestan; HD, Hamadan; HG, Hormozgan; IL, Ilam; KB, Kohgiluyeh and Buyer Ahmad; KD, Kordestan; KE, Kerman; KJ, South Khorasan; KS, North Khorasan; KV, Razavi Khorasan; KZ, Khuzestan; LO, Lorestan; MK, Markazi; MN, Mazandaran; QM, Qom; QZ, Qazvin; SB, Sistan and Baluchestan; SM, Semnan; TE, Tehran; WA, West Azarbaijan; YA, Yazd; ZA, Zanjan.

**Figure 5:**
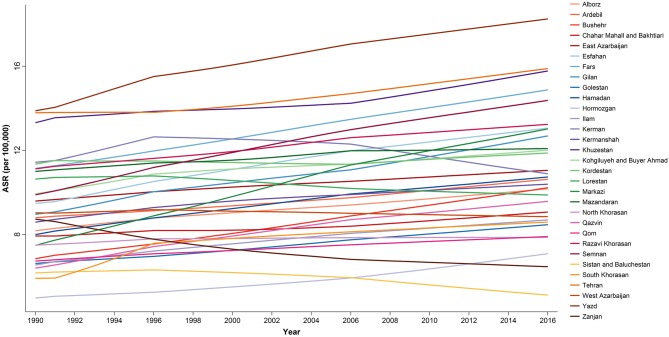
Subnational trend of childhood cancers incidence, 1990–2016.

At the provincial levels, highest ASR in children under 15 belonged to Yazd both in 1990 and 2016 with 13.88 (9.49–20.31) and 18.25 (15.36–21.68) per 100,000 children, respectively (Table 3, Figure S2). Not only the trend of cancer incidence rate was increasing in most of the provinces during the study period (Figure S1), but also the difference between provinces with highest and lowest incidence rates raised by more than 40% during this 27-year study (Figures 4, 5). The relative incidence rate of each region to the total population had increased in regions with highest rate and decreased in regions with lowest incidence rates. Similar to national age-specific patterns, children 10–14 years old had the highest incidence rates in all provinces (Figure S1). The geographical disparity between areas of highest and lowest incidence rates was worst for children 10–14 years old (~50%).

**Table 3:** Provincial incidence rate of all cancers in 1990 and 2016 by age groups and genders.

**Year**		**1990**				**2016**			
**Age**	**Sex**	**Lowest ASR**		**Highest ASR**		**Lowest ASR**		**Highest ASR**	
		**Province**	**ASR (95% UI)**	**Province**	**ASR (95% UI)**	**Province**	**ASR (95% UI)**	**Province**	**ASR (95% UI)**
Under 15	Both	Hormozgan	5 (2.4–10.4)	Yazd	13.9 (9.5–20.3)	Sistan and Baluchestan	5.1 (4.2–6.1)	Yazd	18.1 (15.3–21.6)
	Female	Hormozgan	4.3 (2.1–9.1)	Tehran	12 (7.3–19.8)	Sistan and Baluchestan	4.4 (3.7–5.2)	Yazd	15.8 (13.3–18.8)
	Male	Hormozgan	5.6 (2.7–11.7)	Yazd	15.7 (12.7–22.9)	Sistan and Baluchestan	5.7 (4.8–6.9)	Yazd	20.4 (17.2–24.3)
Under 1	Both	Hormozgan	3.7 (1.8–7.6)	Yazd	10.3 (7.1–15.1)	Sistan and Baluchestan	4 (3.3–4.8)	Yazd	13.9 (11.7–16.6)
	Female	Hormozgan	3.2 (1.5–6.7)	Yazd	9 (6.1–13.1)	Sistan and Baluchestan	3.4 (2.9–4.1)	Yazd	11.7 (9.9–13.9)
	Male	Hormozgan	4.1 (2–8.5)	Yazd	11.6 (7.9–17)	Sistan and Baluchestan	4.5 (3.8–5.4)	Yazd	16 (13.4–19.1)
1–4	Both	Hormozgan	3.9 (1.9–8.1)	Yazd	11.3 (7.7–16.5)	Sistan and Baluchestan	4.2 (3.5–5)	Yazd	15.1 (12.7–18)
	Female	Hormozgan	3.4 (1.6–7)	Yazd	9.7 (6.7–14.3)	Sistan and Baluchestan	3.6 (3–4.3)	Yazd	13.1 (11–15.6)
	Male	Hormozgan	4.4 (2.1–9.1)	Yazd	12.7 (8.7–18.7)	Sistan and Baluchestan	4.8 (4–5.7)	Yazd	17 (14.3–20.3)
5–9	Both	Hormozgan	5.2 (2.5–10.9)	Yazd	14.8 (10.1–21.6)	Sistan and Baluchestan	5.4 (4.5–6.4)	Yazd	19 (16–22.5)
	Female	Hormozgan	4.6 (2.2–9.5)	Yazd	12.8 (8.7–18.7)	Sistan and Baluchestan	4.7 (3.9–5.6)	Yazd	16.6 (14–19.7)
	Male	Hormozgan	5.9 (2.8–12.3)	Yazd	16.7 (11.4–24.4)	Sistan and Baluchestan	6.1 (5.1–7.3)	Yazd	21.3 (17.9–25.3)
10–14	Both	Hormozgan	6.4 (3–13.3)	Tehran	17.1 (10.4–28.1)	Sistan and Baluchestan	6.2 (5.2–7.4)	Yazd	22.3 (18.9–26.4)
	Female	Hormozgan	5.6 (2.7–11.6)	Khuzestan	14.9 (9.9–22.4)	Sistan and Baluchestan	5.3 (4.4–6.4)	Yazd	19.6 (16.5–23.2)
	Male	Hormozgan	7.1 (3.4–14.9)	Tehran	19.2 (11.6–31.5)	Sistan and Baluchestan	7.1 (5.9–8.5)	Yazd	25 (21.1–29.5)

*ASR, age-standardized incidence rate per 100,000; UI, uncertainty interval*.

**Figure 6:**
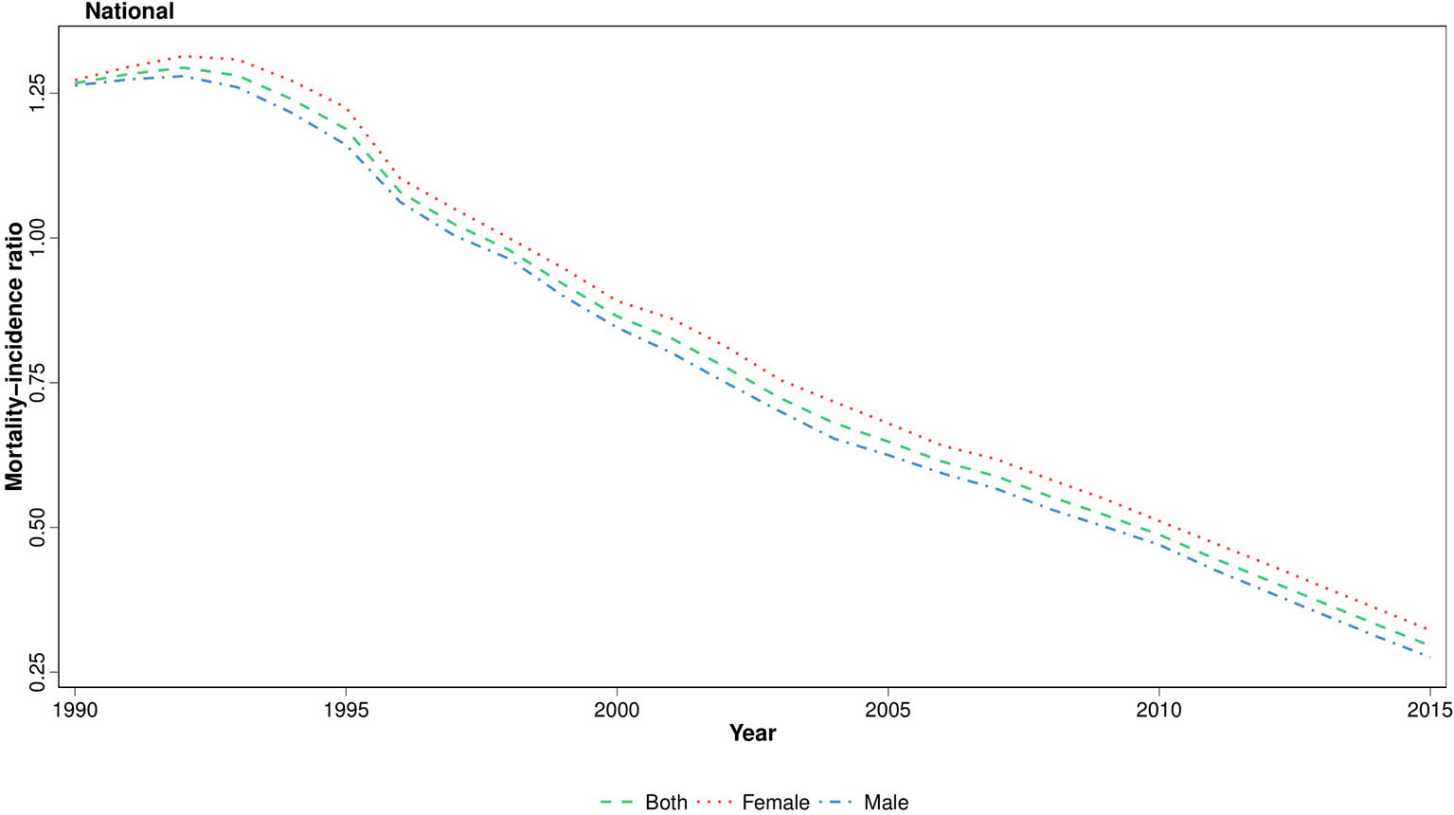
National trend of childhood cancers mortality/incidence ratio (MIR), 1990–2015.

To identify the disparities between cancer diagnosis and treatment, we have calculated the MIR at national and provincial levels, using age-standardized mortality and incidence rates. We observed that the national MIR in 2015 declined remarkably to about one-quarter of the values of 1990 (Figure 6). This descending pattern was observed in all age groups and both genders. Moreover, when analyzing at provincial levels, the difference between the highest and lowest MIR values decreased considerably in 2015 (0.4) compared to 1990 (3.2). Contrary to ASR, there was a female predominance in MIR in more than half of the province-year points (Figure S3). However, at subnational level, the absolute gender difference decreased considerably during the study period (Figure S3).

## Discussion

Herein, we reported the national and subnational trends of childhood cancer incidence from 1990 to 2016. Not only there was a steadily rising incidence of cancers at both levels, but also there was a remarkable progressing subnational disparity. However, in spite of the inclining pattern of ASR, MIR considerably decreased at both levels. Contrary to the expanding gap of ASR among provinces, the difference between MIRs decreased significantly. According to our results, leukemia, cancers of CNS origin, lymphoma, cancers of endocrine glands, digestive organs, and urinary tract were the leading subclasses of childhood cancer.

According to the Global Cancer Observatory, Iran ranks sixth regarding the highest rate of childhood cancers in Asia with an ASR of 13.6 per 100,000 (28). Based on the Institute for Health Metrics and Evaluation, Iran is the fifth country in the Middle East and North Africa region in decreasing order of cancer incidence rate, given a 1.6 per 100,000 incline from 1990 to an incidence rate of 11.05 per 100,000 in 2016 (8). Our data supported the ASR growth in the Institute for Health Metrics and Evaluation. However, the trends of incline were quite different. Despite a constant slight increase in incidence rate observed in our study, the incidence of cancer in children under 20 had an initial slight decline, followed by an incline with a sharper slope toward 2016 (8). Similar patterns were observed in a 10-year study comparing different geographical areas globally, in which all-cause cancer incidence rate increased in all global regions (29). The high incidence rate of cancers of genital origin observed in female arises from the fact that germ cell tumors, a relatively common cancer in children, are recorded under this category. Studies including Surveillance, Epidemiology, and End Results registries similarly report a high contribution of germ cell tumors among childhood cancers (30).

A 34-year (1974–2008) literature review of Iranian childhood cancer incidence reported an incidence rate of 4.8–11.2 and 5.1–14.4 per 100,000 female and male children, respectively (31). Even though the periods are not equivalent, there is not a significant difference in ASR values of our data and the available literature. Moreover, due to the subnational data registries, the number of data points employed in our analysis was several folds more than previous studies. None of the available studies has addressed the subnational level data, and the sources of the global databases discussed have degrees of incompleteness, which makes the generalization of results less conclusive.

Available data on Iranian population dynamics in Iranian census reported a remarkable decline in the proportion of children under 15 to the total population from 39.5% (23,725,545) in 1986 to 24% (19,192,665) in 2016. This declining pattern of growth justifies the declining trend of total new cases diagnosed with cancer during our study period, to some extent. Nevertheless, decomposition analysis showed that changes attributable to increased incidence are still positive, and therefore, there is an excess increasing rate of cancer incidence rate not explicable by the changes in population growth or age structure. This incline may be partly attributable to the progressions in medical care coverage and, consequently, registration of cancer patients (32). Another reasonable explanation for the ascending incidence rate would be the increased awareness in healthcare providers and healthcare seekers (32). This increased awareness may shorten the time before seeking health care and lead to an earlier diagnosis of associated patients.

Our results pointed to a geographical disparity of more than 40% at provincial levels. This is caused by steeper incline in regions with high incidence, probably due to improvements in healthcare coverage resulting in early diagnosis of cancers. On the other hand, provinces with lower ASR have shortage of pediatric oncology specialists and specialized health centers (33). The latter predisposes residents of low-incident regions to unforeseen circumstances in access to care. They would ignore health seeking and/or quit treatment more probably due to financial problems caused by the cost of antineoplastic drugs, the need for migration and unemployment (34). However, part of this difference might be attributed to health behaviors, level of parents' awareness, exposure to varying amounts of environmental factors, urban/rural ratios, pattern of consanguinity, and genetic variations in oncogenes (32, 35).

Although there is a major paucity of evidence in addressing the responsible risk factors for childhood cancer development, ionizing radiation, and immunosuppressive therapies have been identified as causal factors in development of childhood cancers (36). Although national studies have suggested several possible risk factors for childhood cancers (31), data on the status and distribution of different risk factors in Iran is sparse and not included in cancer registries. Hence, the linkage of the inclining trend of cancer incidence to the associated predisposing factors was beyond the scope of this study.

MIR is a method for assessment of ongoing health policies in national cancer management. Recent studies have confirmed a positive association between calculated national cancer MIRs with health system rankings (37). Our findings revealed that cancer care has improved substantially at both national and subnational levels since 1990. However, this improvement relies to a higher extent on declined mortality rate than incidence, since the age-standardized mortality rate was decreased to about a quarter of values of 1990 (38). Although MIR was declined in subnational levels, there was a substantial discrepancy in MIR between provinces. Aside from differences in healthcare services, awareness of patients' caretakers about maintenance of cancer treatment would explain this disparity, since socioeconomic and education levels are the main determinants of treatment abandonment (5). We also observed a female predominance in MIR in most of the regions in the first half of the study period. This vanishing gender difference in MIR is also explainable by the cultural preferences in our country. Yet, additional inferential studies are necessary to prove the association of each determinant.

Distribution of diseases in developing countries is undergoing a transition to non-communicable diseases (8). Thereby, health system policies must provide accommodations compatible with effective management of burdensome causes like cancer. First, we need to assign specialized systems for operating a complete widespread cancer registry, which would be the leading material for designing effective health policies. Regarding the increasing pattern of cancer incidence and the spatiotemporal disparity observed in our study, the establishment of fundamental etiologic researches to identify underlying risk factors of childhood cancers and their distribution at subnational levels seems mandatory. In this regard and given that retrospective studies may bring about biased results in cancer studies, one solution would be to include records of the probable risk factors in national cancer registries.

Fortunately, part of the increasing incidence of recorded cancers might be attributable to the improvements in cancer registries and improvements in diagnostic guidelines. However, since the age of diagnosis of cancer influences the conditional survival of cancer patients (39), developing reasonable yet consentaneous guidelines for earlier screening of cancer in children would lower the attributable mortality rate and increase their quality of life. Albeit, considering the rarity of childhood cancers, conducting screening programs in general population is not economically recommended. Thereby, targeting high-risk populations for cancer screening would be a cost-effective solution. In this regard, American Association for Cancer Research has published a set of screening protocols for genetic syndromes predisposing children to cancers (40–45).

We can claim that, in the case of developing countries, nothing can influence the diagnosis, treatment, and survival of childhood cancers better than educating caregivers about the nature and chronicity of cancers and the essential need for maintaining treatment course. The latter accompanied by training skilled health-care providers and specialized health centers would considerably increase the survival of children with cancer by lowering the MIR (46–48).

Similar to the geographical disparity observed between low, low-middle, and high income countries (49), the subnational disparities in our analysis could be of note for health policy makers in resource and budget allocation among provinces according to their efficacy of cancer management programs reflected in MIR and their individual pattern of population growth.

Finally, as we have noticed in this study, the most noteworthy issue in childhood cancers is the decline in mortality rates despite the incline in incidence rates reflected in decreased MIR values. This finding implies that we have made considerable progresses in diagnostic and therapeutic plans of childhood cancers. However, this is only the tip of an iceberg. We must not miss the dramatic burden of disabilities developed in childhood cancer survivors underneath. Either psychosocial, cognitional, emotional dysfunction, or educational and occupational deprivation of affected individuals causes decreased quality of life and increased disability-adjusted years (6, 50, 51). In addition, a high proportion of families with cancer patients are forced to change their place of residence and jobs and undergo financial issues during the treatment course of their children (52). Thus, provision of supportive programs for patients and their families is pivotal as much, if not more, as developing cancer diagnosis and treatment plans (53, 54).

## Strengths and Limitations

The dramatic burden of cancer is still a health challenge in developing countries, whereas progression in survival care in developed countries have resulted in drastic changes to cure childhood cancers (55). The initial undeniable step in optimizing health policies in childhood cancers is to conduct an adducible population-based study to find under-registered regions, and age or gender groups with the highest level of oversight. As discussed previously, there is no national report of childhood cancer in Iran, and most studies are either local or study a smaller sample of patients. We claim that this national cancer database outweighs other national reports in addressing the incompleteness of cancer registry. To our knowledge, this study is the first to report the trend of national childhood cancer incidence in Iran. In this paper, we reported for the first time a subnational pattern of childhood cancer incidence by means of which the geographical inequalities are concluded. Further assessment of cancer management in subnational levels was done by evaluating MIR among different provinces.

Howsoever, we have confronted several limitations of note. First, this model cannot represent each specific cancer subtype incidence precisely as they are considered rare diseases, and this may affect interpreting the variations in trends. Second, we believe that since various cancer types harbor different etiologic profiles, deployment of the distribution of associated risk factors in models would have improved the estimations. Moreover, even though we have addressed the incompleteness by SSI registry, the data of a small proportion of patients not supported by SSI are still missing in our models. Finally, since we could not capture the incompleteness rate at the subnational level, there might be under-registration in certain provinces. Although we report higher completeness, there is yet to be done to reach a full-coverage cancer registry.

## Conclusion

Studying the health demands of a population is mandatory for establishing strategies to control burdensome causes. We have reported an increasing pattern of incidence rate despite a tremendous decline in mortality rate due to childhood cancers. Even though similar alterations were observed in most of the provinces and all age groups, we noticed a remarkable progressing gap in cancer incidence but not cancer mortality at subnational levels. In this regard, it is pivotal to address the distribution of possible risk factors and their association with variable cancer incidence, probably by improving the national cancer registries. Meanwhile, conducting large-sample nested studies among diverse geographic regions and risk factor monitoring would be helpful. Moreover, part of this disparity is related to delayed or missed diagnosis in regions with low incidence, which might be due to the low awareness of caretakers and lack of specialized caregivers and health centers. Educating caregivers about the symptoms and the chronic nature of the disease and providing specialized health workers and pediatric hospitals would cause dramatic improvements in the management of childhood cancers. We believe that employment of available data on the distribution of cancer incidence and mortality in this study for resource allocation and policy making by regional needs may evolve the childhood cancer care in national, regional, and global levels.

## Data Availability Statement

All datasets generated for this study are included in the article/Supplementary Material.

## Ethics Statement

The Ethics Committee of the National Institute for Medical Research Development approved the study protocol (IR.NIMAD.REC.1396.192).

## Author Contributions

MS: conceptualization, investigation, project administration, validation, and writing. SS: data curation, formal analysis, methodology, and software. BA: investigation, formal analysis, validation, and writing. NR: conceptualization, funding acquisition, project administration, and supervision. FM and BM: resources, data curation, and validation. KG: data curation, software, and formal analysis. AS and FP: methodology, data curation, and resources. MY: data curation, methodology, and software. FK: conceptualization, project administration, resources, supervision, and writing. FF: conceptualization, funding acquisition, methodology, validation, and writing.

### Conflict of Interest

The authors declare that the research was conducted in the absence of any commercial or financial relationships that could be construed as a potential conflict of interest. The handling Editor declared a past co-authorship with one of the authors FF.
