# Supplementary material for: Trends of National and Subnational Incidence of Childhood Cancer Groups in Iran: 1990–2016
Source: Front Oncol. 2020 Jan 14;9:1428. doi: 10.3389/fonc.2019.01428 (PMC6970968; doi:10.3389/fonc.2019.01428)
Supplement: Supplementary file 1 [file Data_Sheet_1.PDF]

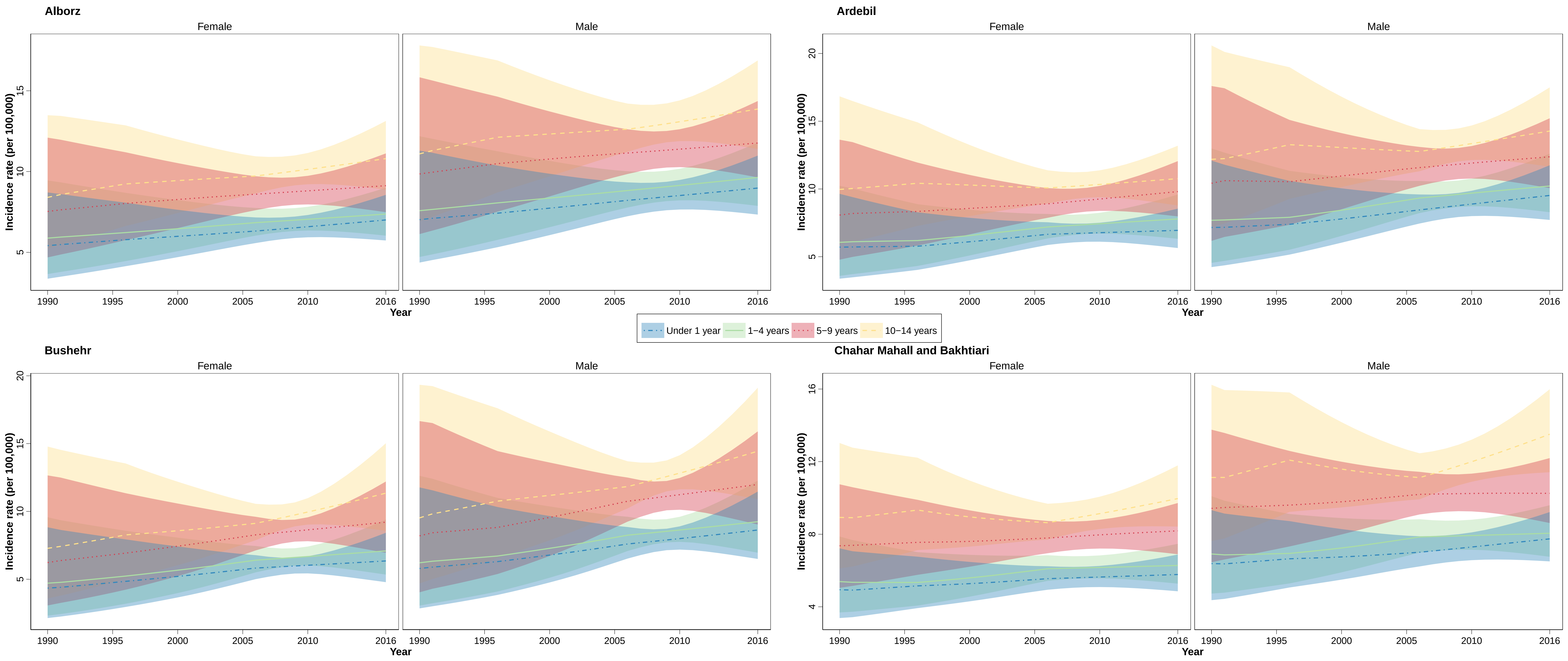
Alborz
Ardebil
Bushehr
Chahar Mahall and Bakhtiari
Female
Male
Incidence rate (per 100,000)
Year
Under 1 year
1−4 years
5−9 years
10−14 years

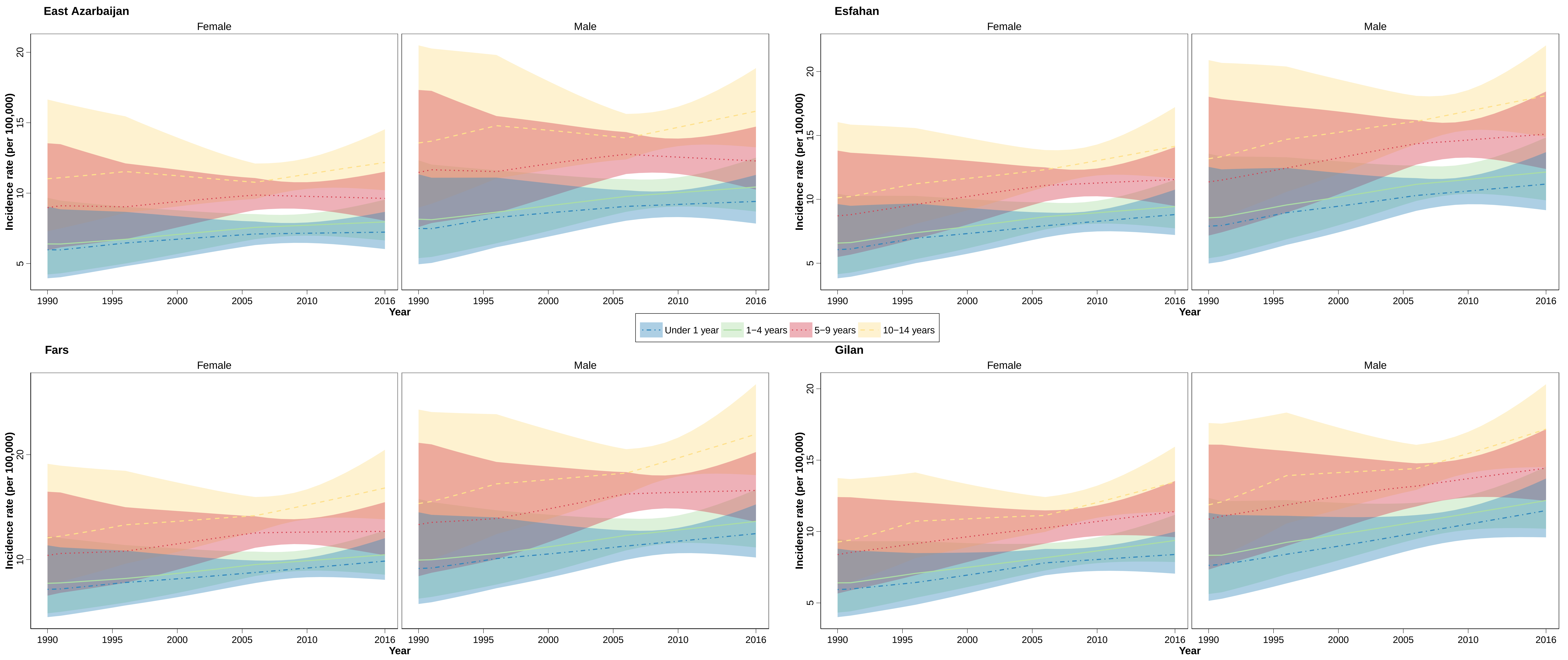

East Azarbaijan
Esfahan
Fars
Gilan
Female
Male
Incidence rate (per 100,000)
Year
1990
1995
2000
2005
2010
2016
5
10
15
20
Under 1 year
1–4 years
5–9 years
10–14 years

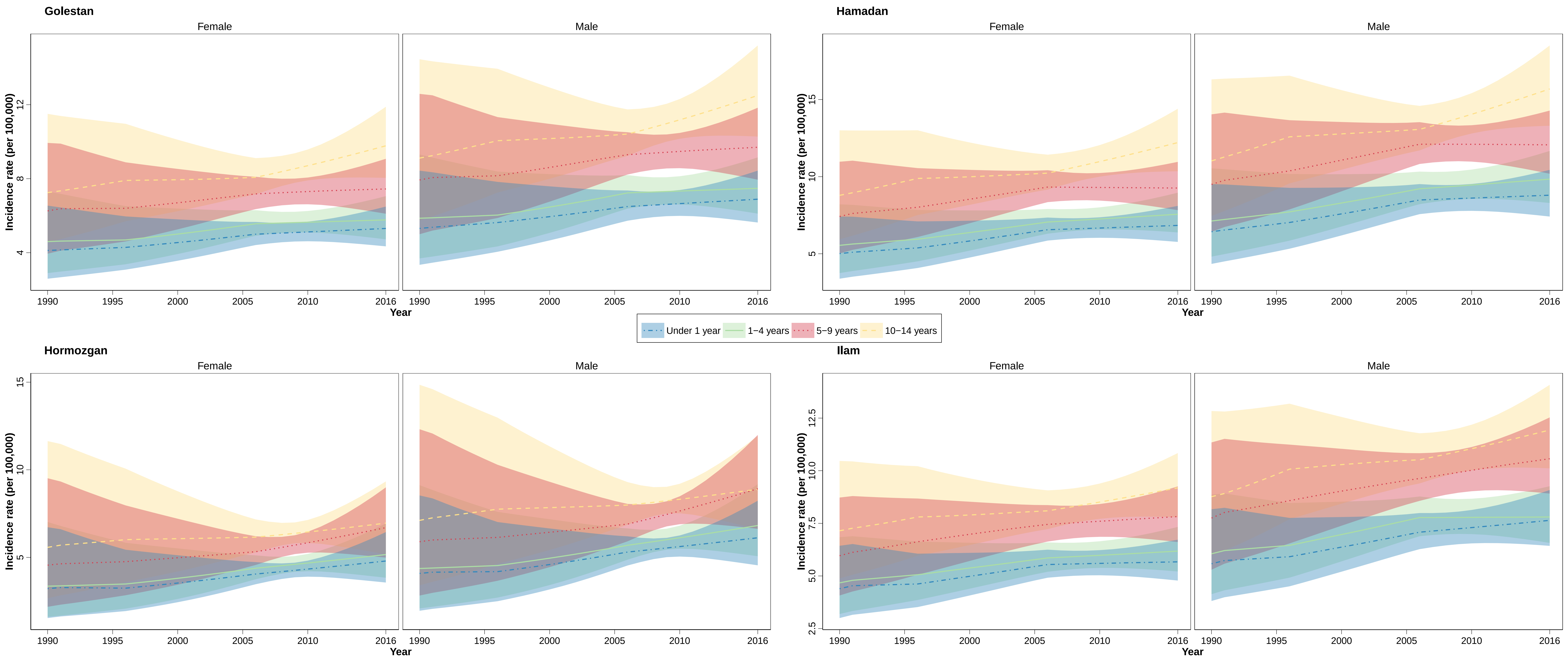

Golestan
Hamadan
Hormozgan
Ilam
Female
Male
Incidence rate (per 100,000)
Year
1990
1995
2000
2005
2010
2016
4
8
12
5
10
15
2.5
5.0
7.5
10.0
12.5
Under 1 year
1–4 years
5–9 years
10–14 years

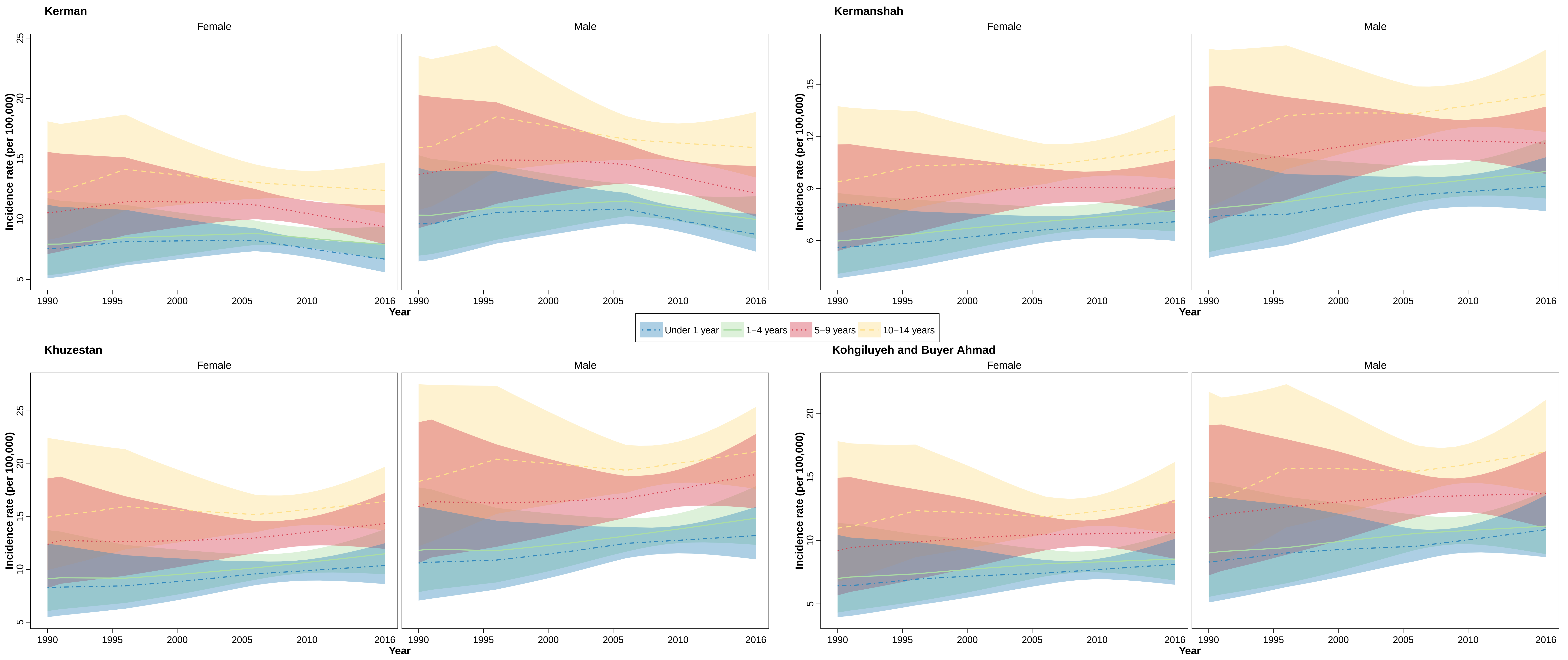
Kerman
Kermanshah
Khuzestan
Kohgiluyeh and Buyer Ahmad
Female
Male
Incidence rate (per 100,000)
Year
Under 1 year
1−4 years
5−9 years
10−14 years

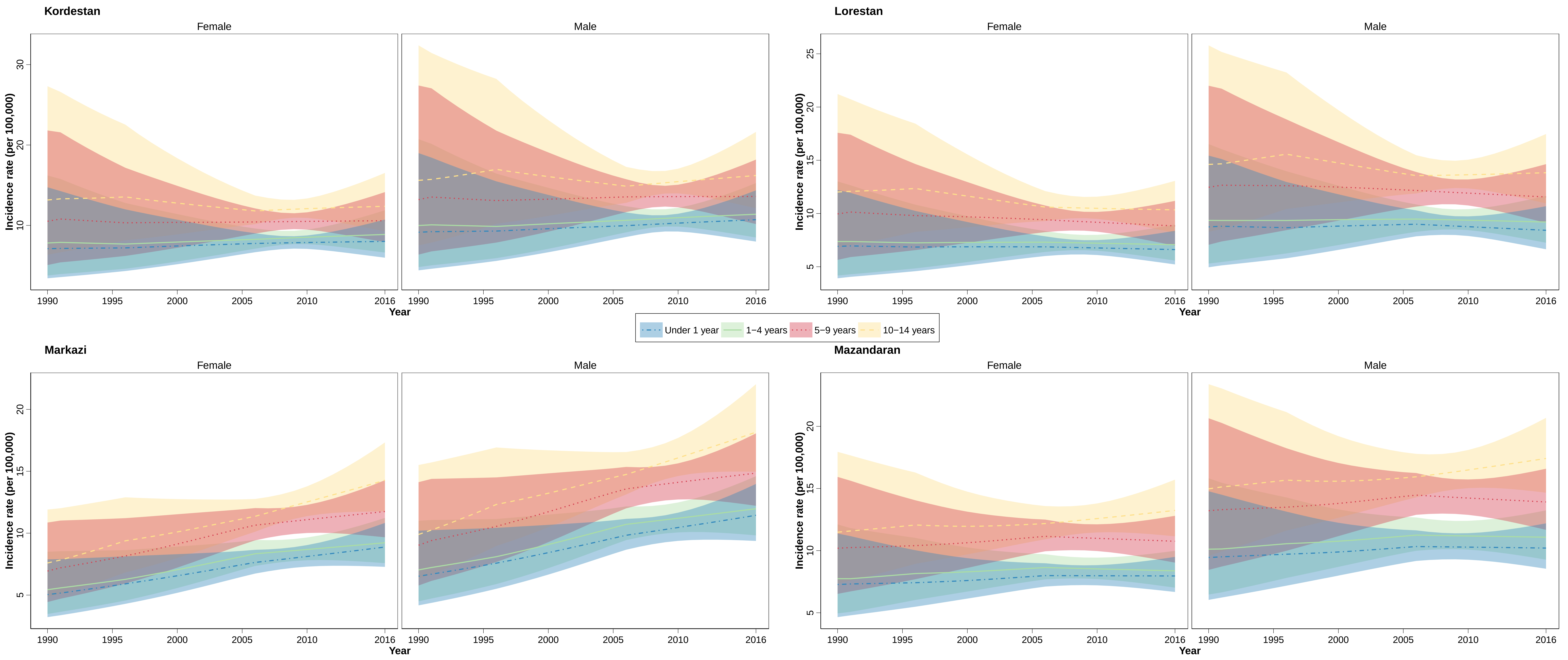

Kordestan
Lorestan
Markazi
Mazandaran
Female
Male
Incidence rate (per 100,000)
Year
Under 1 year
1–4 years
5–9 years
10–14 years

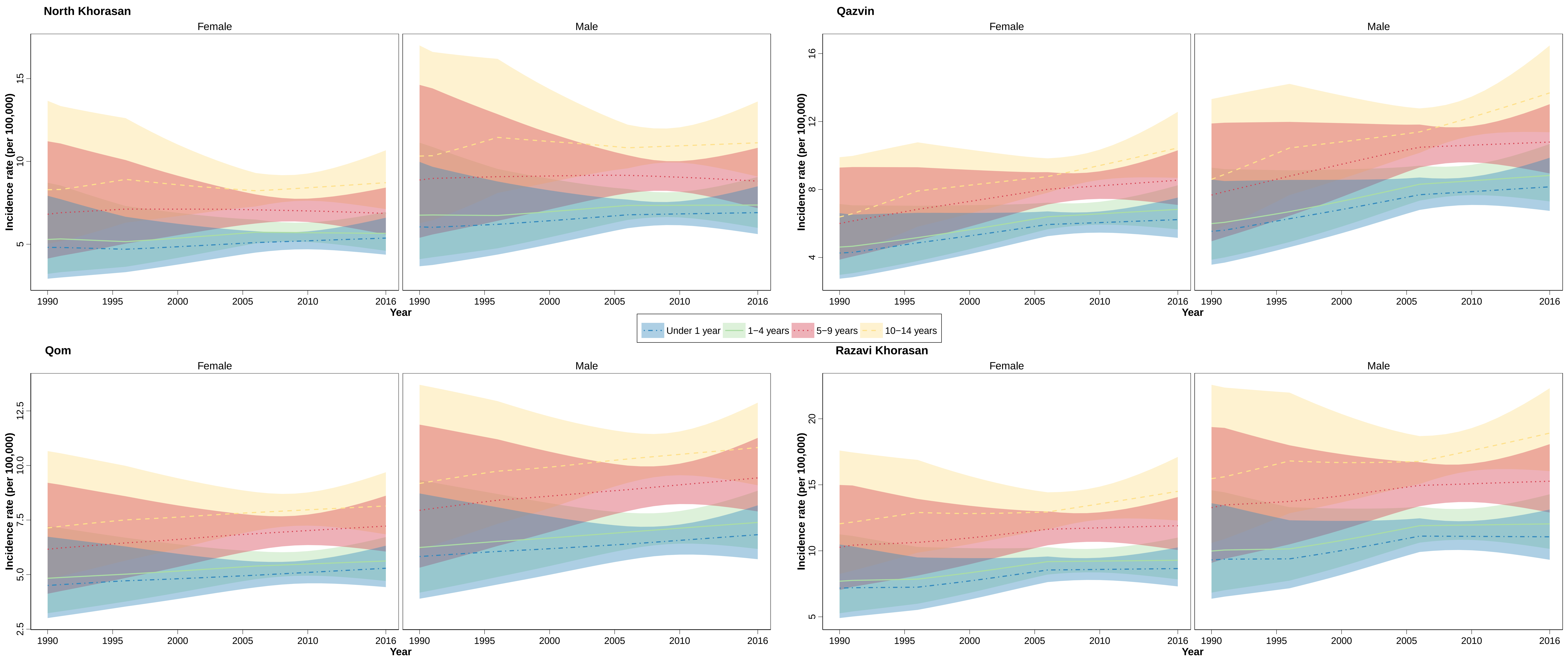

North Khorasan
Qazvin
Qom
Razavi Khorasan
Female
Male
Incidence rate (per 100,000)
Year
Under 1 year
1−4 years
5−9 years
10−14 years

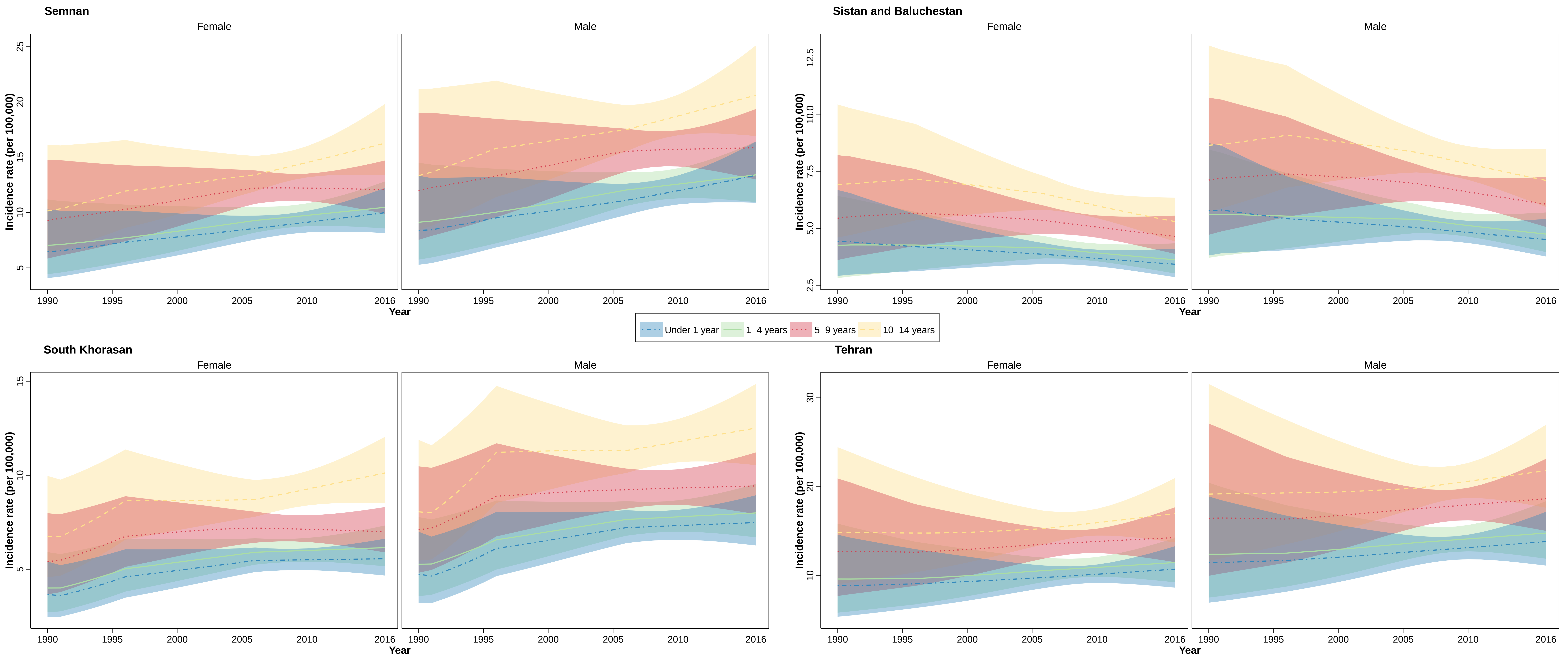

Semnan
Sistan and Baluchestan
South Khorasan
Tehran
Female
Male
Incidence rate (per 100,000)
Year
1990
1995
2000
2005
2010
2016
Under 1 year
1–4 years
5–9 years
10–14 years

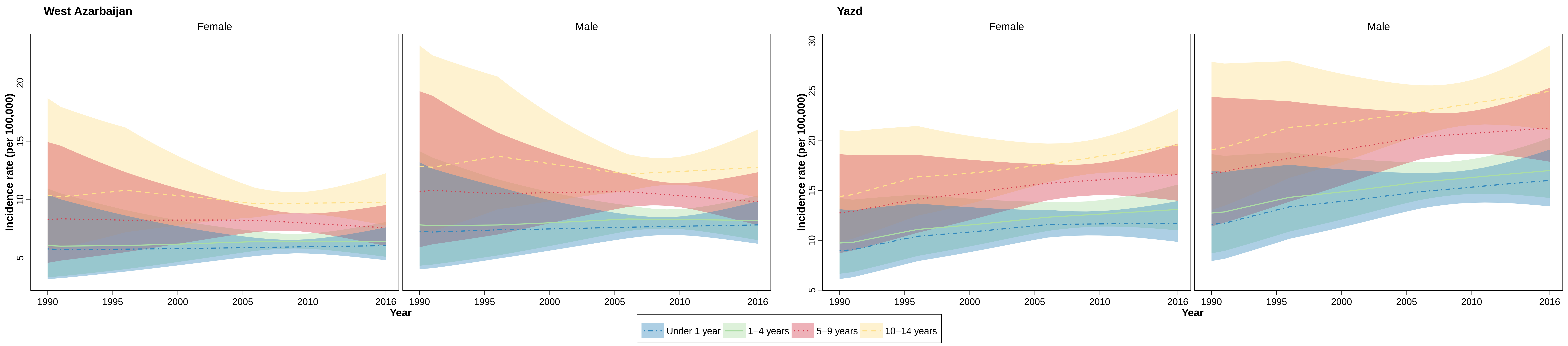

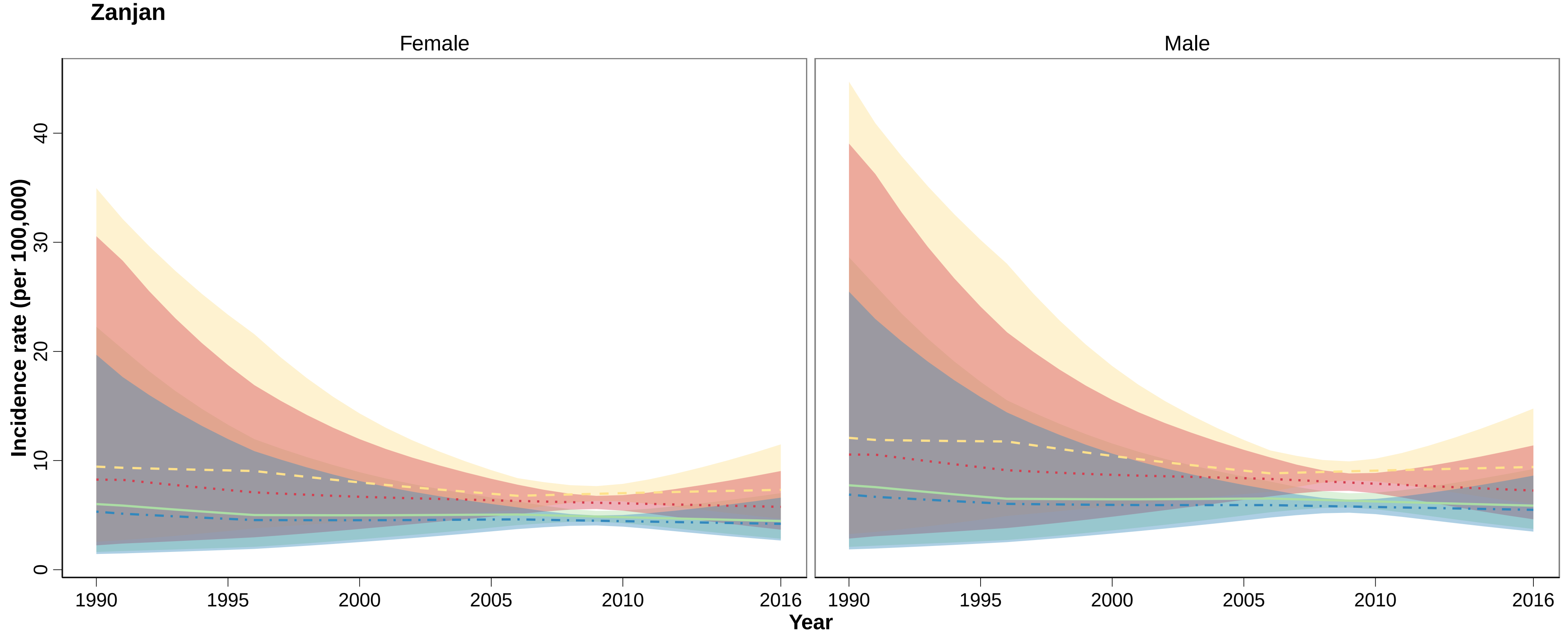

FIGURE S1 | Time trend of childhood cancer incidence by four major age groups at subnational level. Incidence rate of all-cause cancers depicted in lines for under 1 year (blue), 1–4 years (green), 5–9 years (red), and 10–14 years (yellow). The colored areas shows the 95% uncertainty interval for the associated lines.
