# Supplementary figures and images for: Trends of National and Subnational Incidence of Childhood Cancer Groups in Iran: 1990–2016
Source: Front Oncol. 2020 Jan 14;9:1428. doi: 10.3389/fonc.2019.01428 (PMC6970968; doi:10.3389/fonc.2019.01428)

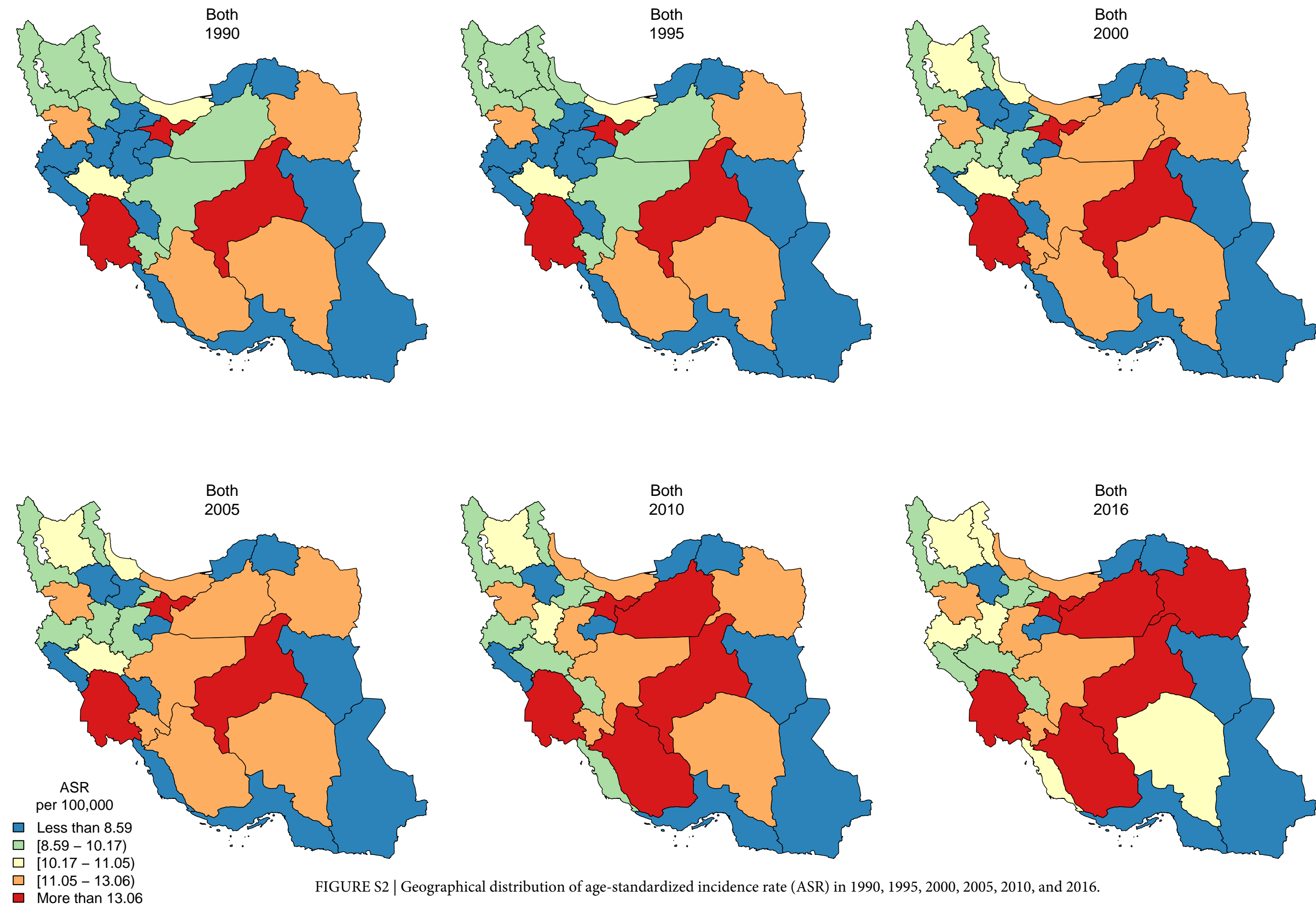

Supplement: Supplementary file 2 [file Data_Sheet_2.PDF]
