# Supplementary material for: Trends of National and Subnational Incidence of Childhood Cancer Groups in Iran: 1990–2016
Source: Front Oncol. 2020 Jan 14;9:1428. doi: 10.3389/fonc.2019.01428 (PMC6970968; doi:10.3389/fonc.2019.01428)
Supplement: Supplementary file 3 [file Data_Sheet_3.PDF]

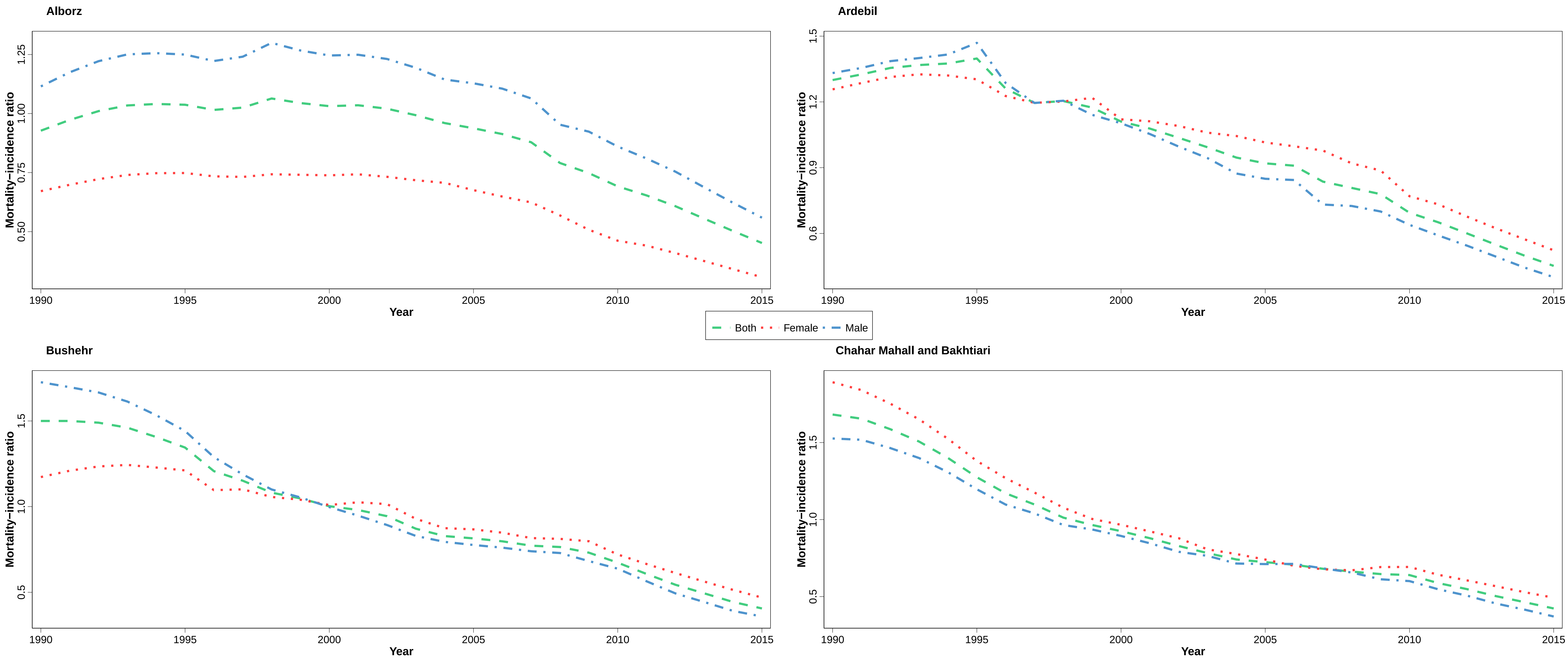
Alborz
Ardebil
Bushehr
Chahar Mahall and Bakhtiari
Mortality−incidence ratio
Year
1990
1995
2000
2005
2010
2015
0.50
0.75
1.00
1.25
0.6
0.9
1.2
1.5
0.5
1.0
1.5
Both
Female
Male

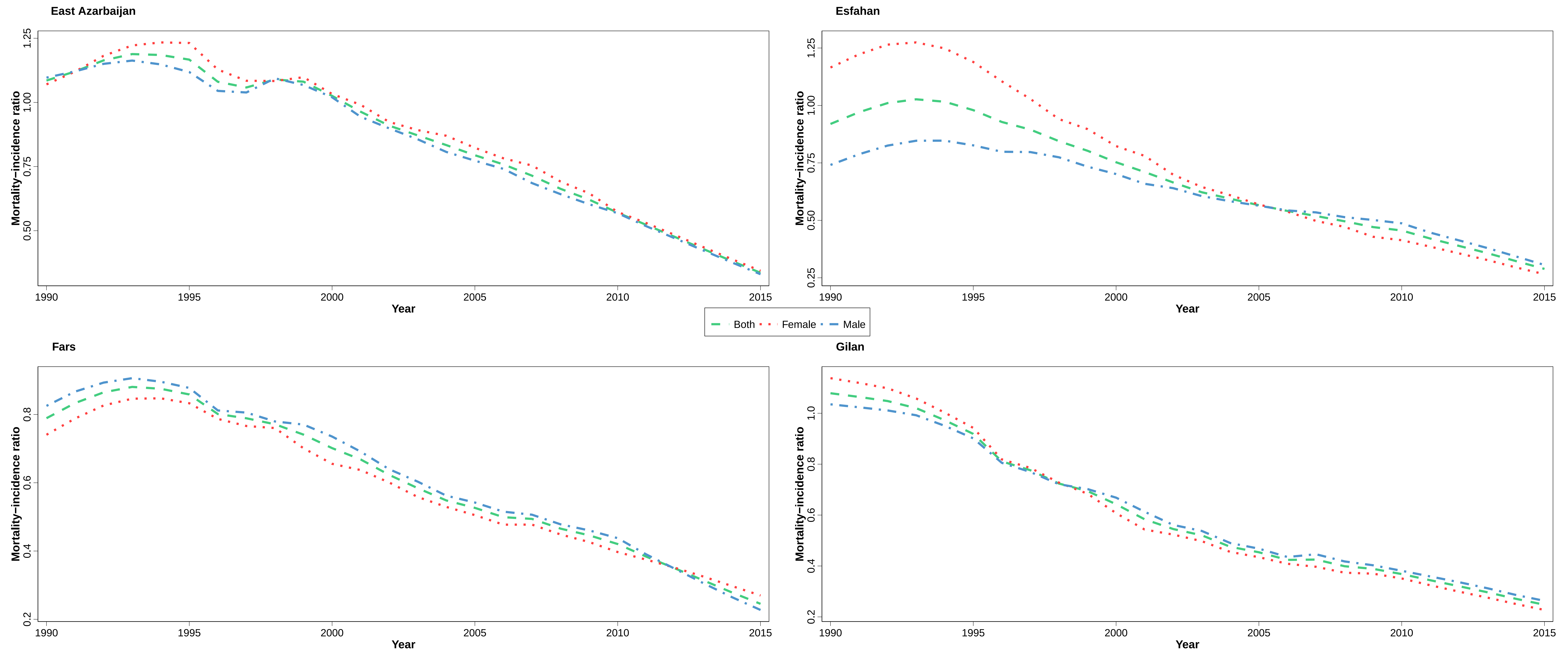
East Azarbaijan
Esfahan
Fars
Gilan
Mortality−incidence ratio
Year
1990
1995
2000
2005
2010
2015
0.25
0.50
0.75
1.00
1.25
0.2
0.4
0.6
0.8
1.0
Both
Female
Male

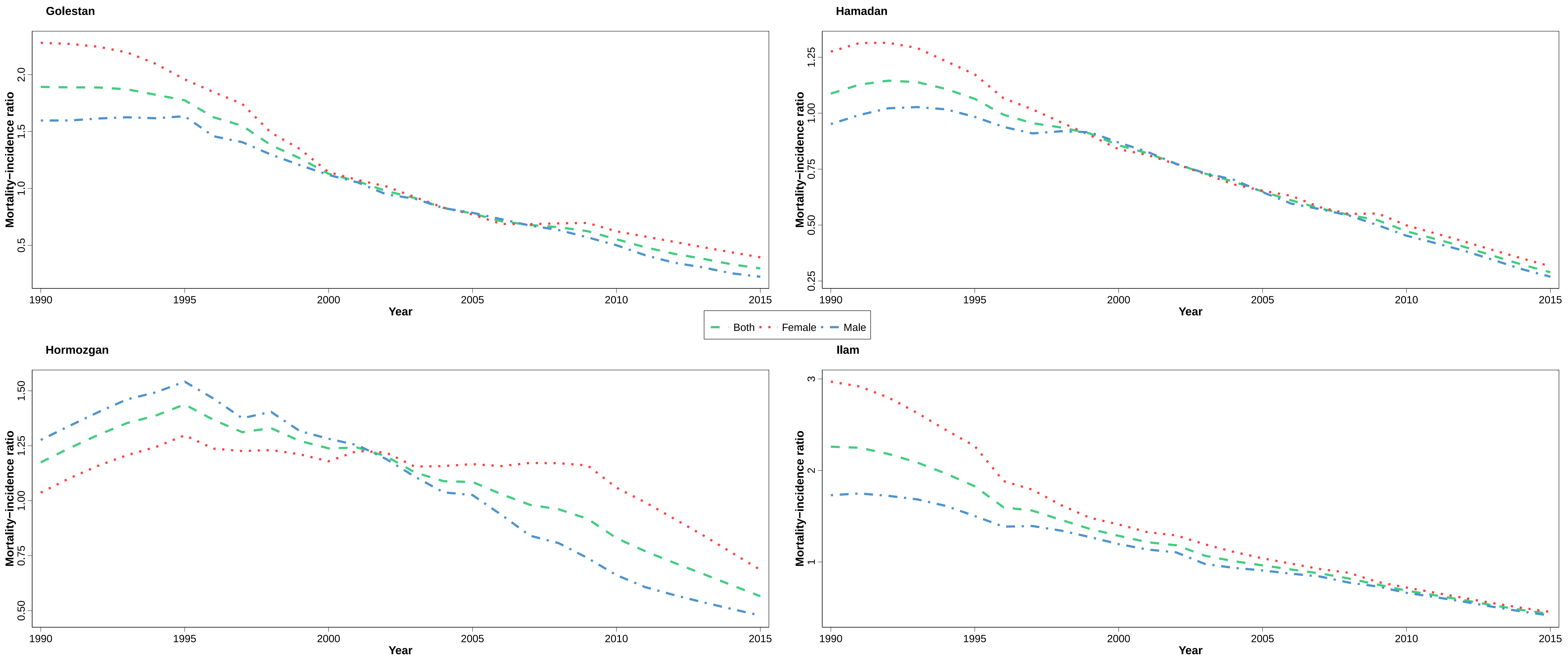

Golestan
Hamadan
Hormozgan
Ilam
Mortality−incidence ratio
Year
1990
1995
2000
2005
2010
2015
0.5
1.0
1.5
2.0
0.25
0.50
0.75
1.00
1.25
1
2
3
Both
Female
Male

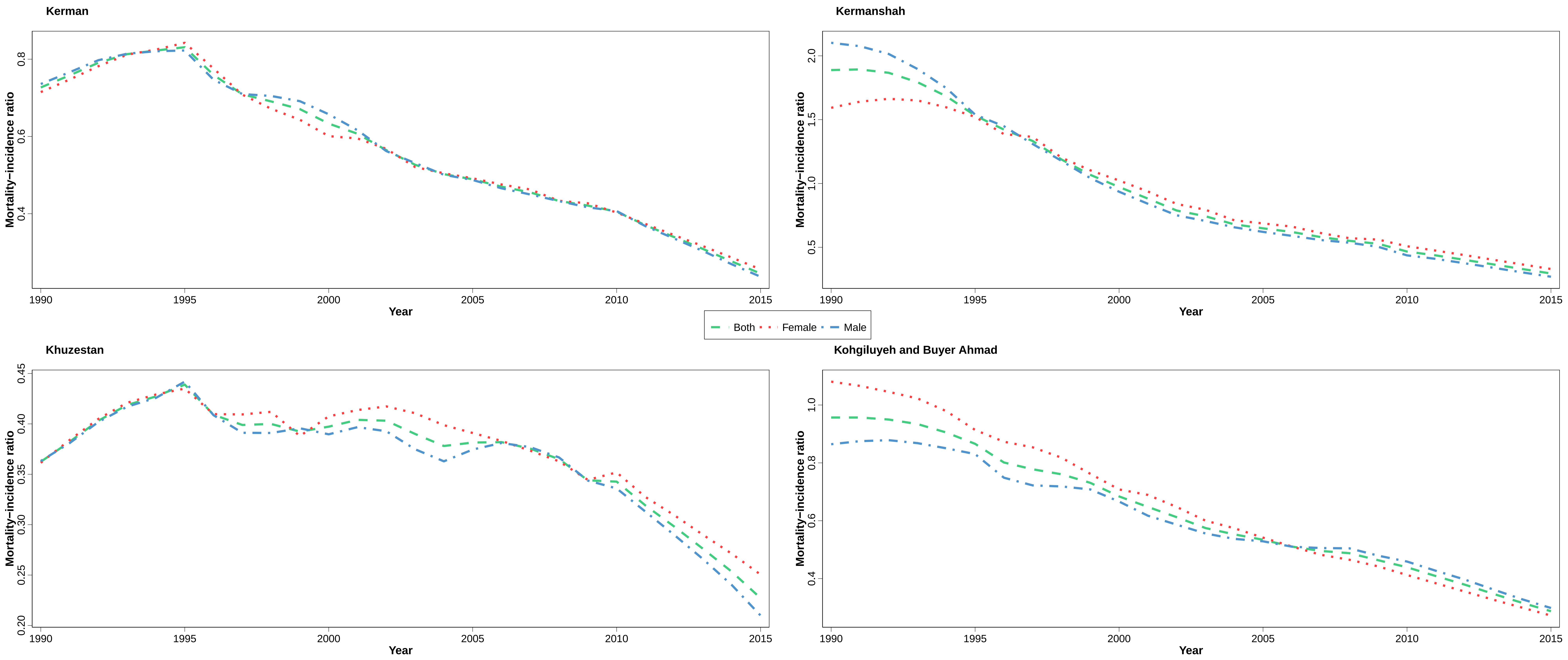

Kerman
Kermanshah
Khuzestan
Kohgiluyeh and Buyer Ahmad
Mortality−incidence ratio
Year
1990
1995
2000
2005
2010
2015
0.4
0.6
0.8
0.5
1.0
1.5
2.0
0.20
0.25
0.30
0.35
0.40
0.45
0.4
0.6
0.8
1.0
Both
Female
Male

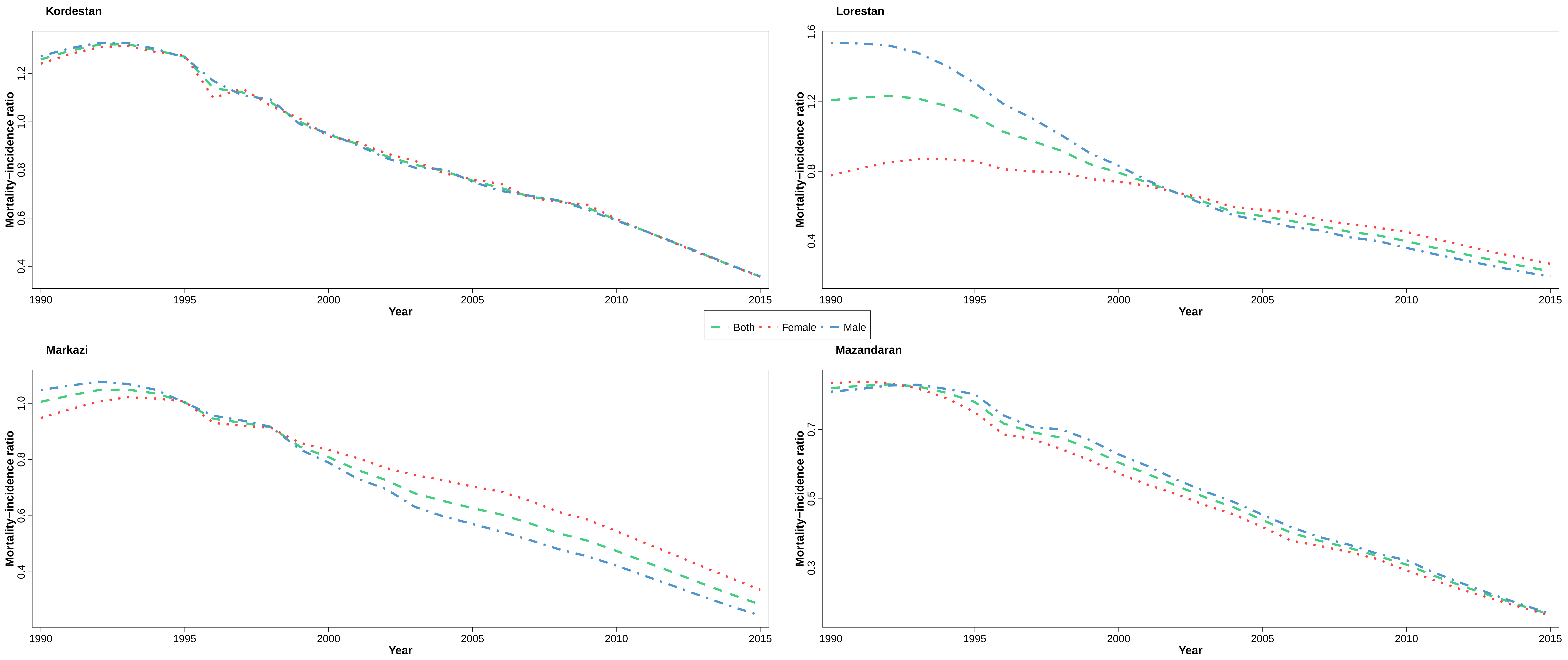

Kordestan
Lorestan
Markazi
Mazandaran
Mortality−incidence ratio
Year
1990
1995
2000
2005
2010
2015
0.4
0.6
0.8
1.0
1.2
1.6
0.5
0.7
0.3
Both
Female
Male

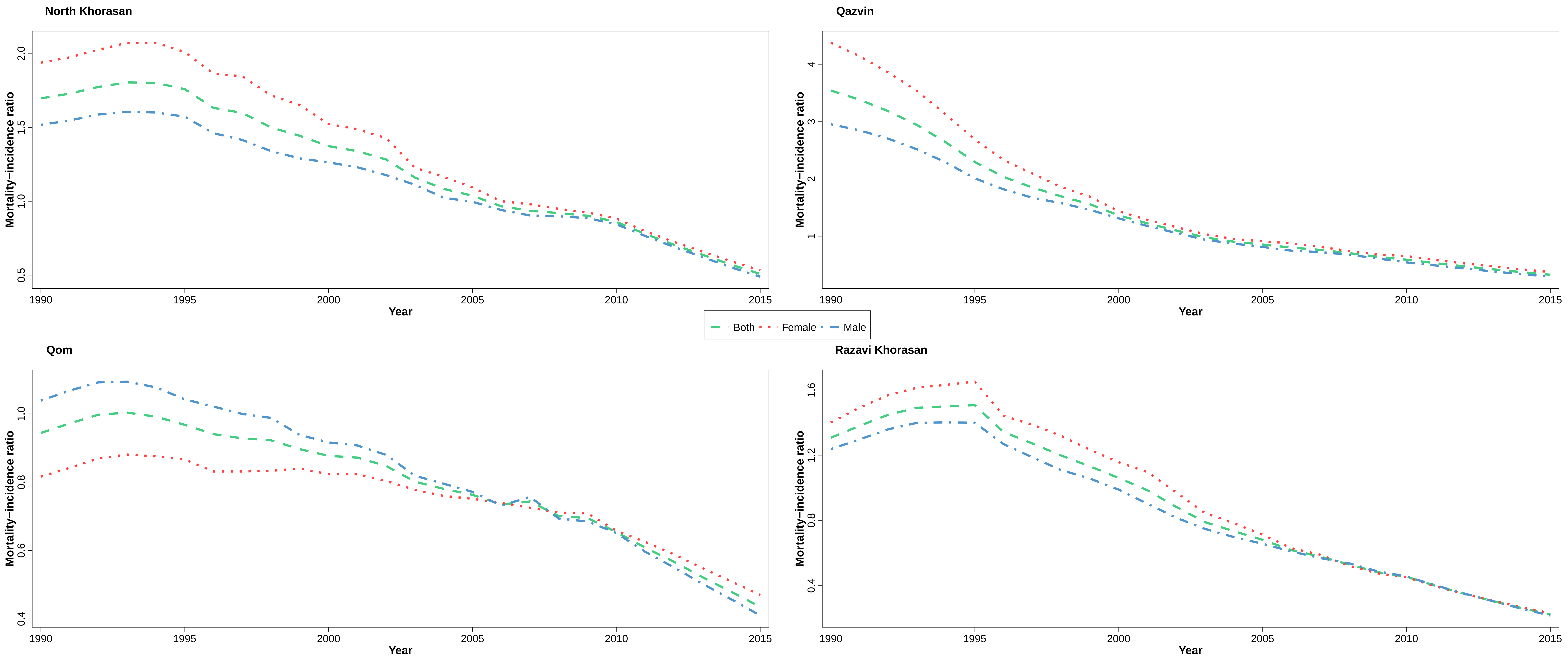

North Khorasan
Qazvin
Qom
Razavi Khorasan
Mortality−incidence ratio
Year
Both
Female
Male
1990
1995
2000
2005
2010
2015

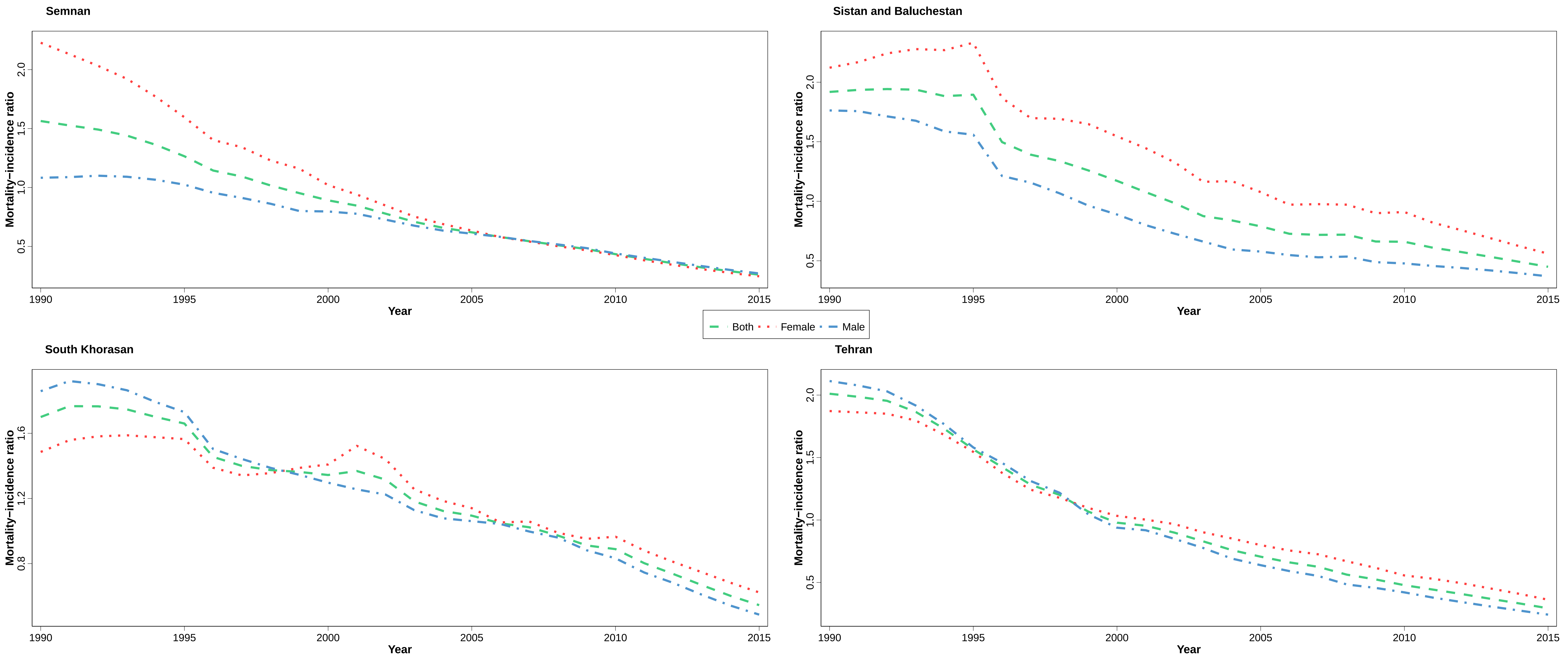

Semnan
Sistan and Baluchestan
South Khorasan
Tehran
Mortality−incidence ratio
Year
Both
Female
Male

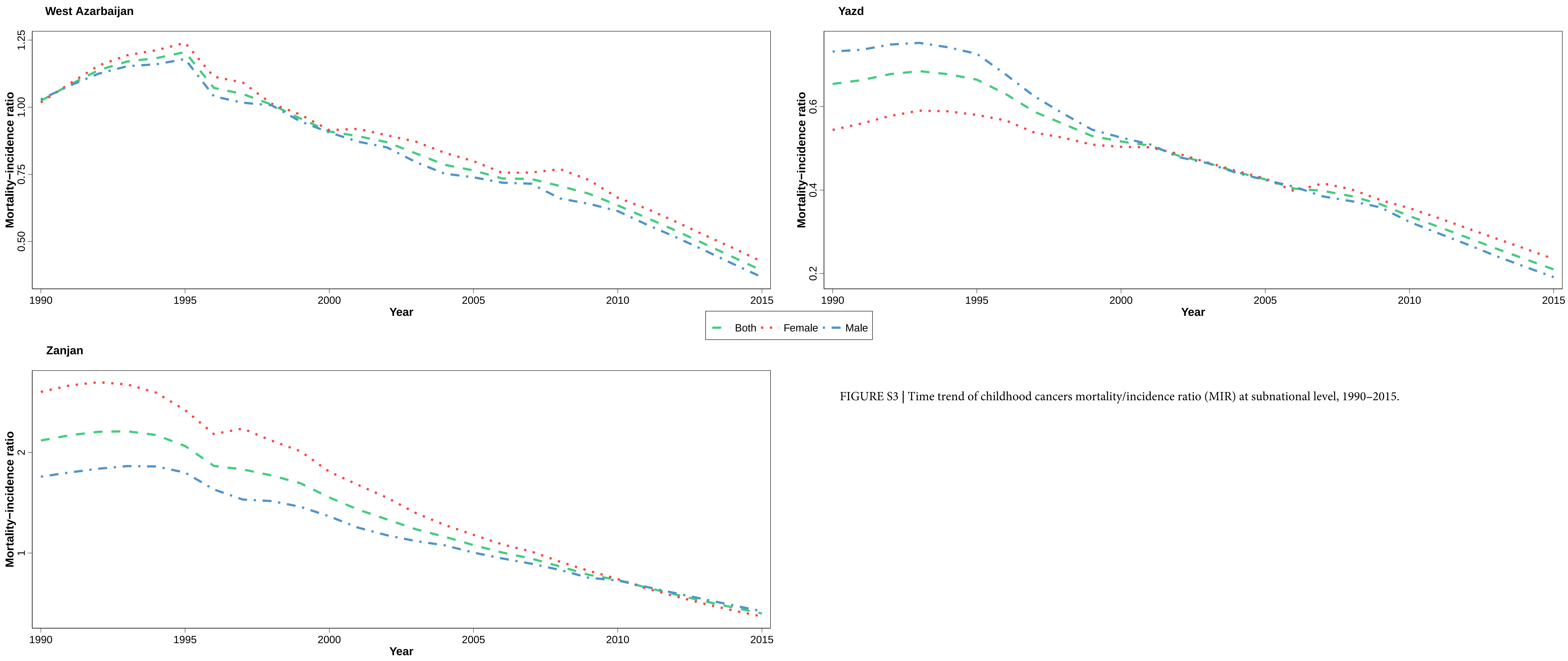

FIGURE S3 | Time trend of childhood cancers mortality/incidence ratio (MIR) at subnational level, 1990–2015.
