# Supplementary material for: Trends of National and Subnational Incidence of Childhood Cancer Groups in Iran: 1990–2016
Source: Front Oncol. 2020 Jan 14;9:1428. doi: 10.3389/fonc.2019.01428 (PMC6970968; doi:10.3389/fonc.2019.01428)
Supplement: Supplementary file 4 [file Data_Sheet_4.PDF]

	Under 15			Under 1			1 to 4			5 to 9			10 to 14		
**Year**	**Both**	**Female**	**Male**	**Both**	**Female**	**Male**	**Both**	**Female**	**Male**	**Both**	**Female**	**Male**	**Both**	**Female**	**Male**
**1990**	10.08 (6.33 to 16.33)	8.81 (5.53 to 14.27)	11.29 (7.09 to 18.28)	7.41 (4.65 to 12.03)	6.47 (4.06 to 10.49)	8.32 (5.22 to 13.49)	8 (5.02 to 12.97)	6.96 (4.37 to 11.29)	9 (5.65 to 14.59)	10.72 (6.74 to 17.35)	9.34 (5.86 to 15.11)	12.05 (7.57 to 19.5)	12.52 (7.86 to 20.28)	11 (6.9 to 17.82)	13.94 (8.76 to 22.59)
**1991**	10.18 (6.54 to 16.08)	8.9 (5.72 to 14.05)	11.41 (7.33 to 18.01)	7.44 (4.78 to 11.77)	6.49 (4.16 to 10.26)	8.35 (5.36 to 13.21)	8.03 (5.16 to 12.69)	6.99 (4.49 to 11.05)	9.03 (5.8 to 14.28)	10.89 (7 to 17.18)	9.48 (6.1 to 14.96)	12.24 (7.87 to 19.31)	12.65 (8.13 to 19.98)	11.1 (7.14 to 17.54)	14.1 (9.06 to 22.26)
**1992**	10.27 (6.75 to 15.82)	8.97 (5.89 to 13.81)	11.51 (7.57 to 17.72)	7.52 (4.94 to 11.59)	6.55 (4.3 to 10.1)	8.43 (5.54 to 13.01)	8.08 (5.31 to 12.46)	7.03 (4.62 to 10.84)	9.09 (5.97 to 14.02)	10.93 (7.19 to 16.82)	9.51 (6.26 to 14.65)	12.28 (8.08 to 18.91)	12.83 (8.44 to 19.76)	11.24 (7.39 to 17.31)	14.33 (9.43 to 22.06)
**1993**	10.35 (6.96 to 15.56)	9.03 (6.08 to 13.58)	11.61 (7.81 to 17.45)	7.59 (5.1 to 11.42)	6.61 (4.44 to 9.94)	8.52 (5.72 to 12.82)	8.14 (5.47 to 12.24)	7.08 (4.76 to 10.65)	9.15 (6.15 to 13.77)	10.96 (7.38 to 16.47)	9.55 (6.42 to 14.35)	12.32 (8.29 to 18.52)	13.02 (8.76 to 19.55)	11.38 (7.66 to 17.09)	14.57 (9.81 to 21.87)
**1994**	10.44 (7.18 to 15.31)	9.1 (6.26 to 13.35)	11.71 (8.06 to 17.18)	7.66 (5.27 to 11.25)	6.67 (4.59 to 9.79)	8.6 (5.91 to 12.63)	8.19 (5.63 to 12.02)	7.12 (4.9 to 10.45)	9.21 (6.33 to 13.52)	11 (7.57 to 16.14)	9.58 (6.6 to 14.05)	12.37 (8.51 to 18.14)	13.2 (9.1 to 19.35)	11.52 (7.94 to 16.89)	14.8 (10.2 to 21.69)
**1995**	10.53 (7.41 to 15.07)	9.17 (6.46 to 13.13)	11.82 (8.32 to 16.92)	7.74 (5.44 to 11.08)	6.73 (4.73 to 9.64)	8.69 (6.11 to 12.45)	8.24 (5.79 to 11.81)	7.16 (5.04 to 10.27)	9.26 (6.51 to 13.28)	11.04 (7.77 to 15.81)	9.62 (6.77 to 13.77)	12.41 (8.73 to 17.77)	13.4 (9.44 to 19.16)	11.67 (8.23 to 16.7)	15.05 (10.61 to 21.52)
**1996**	10.62 (7.64 to 14.84)	9.25 (6.66 to 12.93)	11.93 (8.59 to 16.67)	7.81 (5.62 to 10.92)	6.79 (4.88 to 9.5)	8.77 (6.31 to 12.27)	8.29 (5.96 to 11.61)	7.21 (5.18 to 10.09)	9.32 (6.7 to 13.05)	11.08 (7.98 to 15.49)	9.65 (6.95 to 13.5)	12.45 (8.96 to 17.41)	13.6 (9.8 to 18.98)	11.83 (8.53 to 16.52)	15.3 (11.03 to 21.35)
**1997**	10.67 (7.86 to 14.58)	9.29 (6.84 to 12.69)	11.99 (8.83 to 16.38)	7.89 (5.8 to 10.78)	6.86 (5.04 to 9.37)	8.86 (6.52 to 12.11)	8.39 (6.17 to 11.47)	7.29 (5.36 to 9.97)	9.43 (6.93 to 12.9)	11.17 (8.22 to 15.25)	9.73 (7.16 to 13.28)	12.55 (9.24 to 17.15)	13.57 (10 to 18.5)	11.8 (8.7 to 16.1)	15.27 (11.26 to 20.82)
**1998**	10.73 (8.08 to 14.32)	9.34 (7.03 to 12.47)	12.06 (9.08 to 16.09)	7.97 (5.99 to 10.65)	6.93 (5.21 to 9.25)	8.95 (6.73 to 11.96)	8.48 (6.38 to 11.33)	7.37 (5.54 to 9.85)	9.54 (7.17 to 12.75)	11.25 (8.47 to 15.02)	9.8 (7.38 to 13.07)	12.65 (9.52 to 16.89)	13.54 (10.21 to 18.05)	11.78 (8.87 to 15.7)	15.24 (11.49 to 20.31)
**1999**	10.79 (8.3 to 14.08)	9.39 (7.23 to 12.25)	12.13 (9.33 to 15.82)	8.05 (6.18 to 10.51)	7 (5.38 to 9.13)	9.05 (6.95 to 11.81)	8.58 (6.59 to 11.21)	7.45 (5.73 to 9.73)	9.65 (7.41 to 12.6)	11.34 (8.73 to 14.79)	9.87 (7.6 to 12.87)	12.75 (9.81 to 16.64)	13.52 (10.42 to 17.61)	11.76 (9.06 to 15.32)	15.22 (11.72 to 19.82)
**2000**	10.86 (8.54 to 13.84)	9.45 (7.43 to 12.04)	12.2 (9.59 to 15.56)	8.13 (6.38 to 10.38)	7.07 (5.55 to 9.02)	9.14 (7.17 to 11.67)	8.68 (6.81 to 11.08)	7.54 (5.92 to 9.62)	9.76 (7.66 to 12.47)	11.43 (8.99 to 14.58)	9.95 (7.82 to 12.68)	12.85 (10.1 to 16.39)	13.5 (10.63 to 17.2)	11.74 (9.24 to 14.96)	15.19 (11.96 to 19.34)
**2001**	10.92 (8.77 to 13.62)	9.5 (7.64 to 11.85)	12.27 (9.86 to 15.3)	8.22 (6.59 to 10.26)	7.14 (5.73 to 8.91)	9.24 (7.41 to 11.54)	8.78 (7.04 to 10.96)	7.63 (6.12 to 9.52)	9.88 (7.92 to 12.34)	11.52 (9.26 to 14.37)	10.02 (8.06 to 12.49)	12.96 (10.41 to 16.16)	13.49 (10.86 to 16.8)	11.73 (9.44 to 14.61)	15.17 (12.21 to 18.89)
**2002**	10.99 (9.02 to 13.4)	9.56 (7.85 to 11.66)	12.35 (10.13 to 15.06)	8.3 (6.8 to 10.15)	7.21 (5.91 to 8.81)	9.33 (7.65 to 11.41)	8.89 (7.28 to 10.86)	7.72 (6.33 to 9.42)	10 (8.19 to 12.22)	11.61 (9.53 to 14.16)	10.1 (8.29 to 12.31)	13.06 (10.72 to 15.93)	13.48 (11.08 to 16.41)	11.72 (9.64 to 14.28)	15.16 (12.46 to 18.45)
**2003**	11.05 (9.27 to 13.2)	9.62 (8.06 to 11.48)	12.42 (10.41 to 14.83)	8.39 (7.02 to 10.04)	7.29 (6.1 to 8.71)	9.43 (7.89 to 11.29)	8.99 (7.53 to 10.76)	7.81 (6.54 to 9.34)	10.12 (8.46 to 12.1)	11.7 (9.81 to 13.97)	10.17 (8.53 to 12.14)	13.16 (11.02 to 15.71)	13.47 (11.31 to 16.05)	11.71 (9.84 to 13.96)	15.14 (12.72 to 18.04)
**2004**	11.12 (9.51 to 13.01)	9.67 (8.28 to 11.31)	12.49 (10.68 to 14.62)	8.48 (7.23 to 9.94)	7.36 (6.28 to 8.63)	9.53 (8.13 to 11.18)	9.1 (7.77 to 10.67)	7.9 (6.75 to 9.26)	10.24 (8.74 to 12.01)	11.78 (10.07 to 13.78)	10.24 (8.76 to 11.98)	13.25 (11.33 to 15.51)	13.45 (11.53 to 15.71)	11.71 (10.03 to 13.67)	15.12 (12.96 to 17.65)
**2005**	11.18 (9.74 to 12.84)	9.73 (8.48 to 11.16)	12.57 (10.94 to 14.43)	8.56 (7.44 to 9.86)	7.43 (6.46 to 8.55)	9.63 (8.37 to 11.09)	9.21 (8 to 10.6)	8 (6.95 to 9.19)	10.36 (9 to 11.93)	11.86 (10.33 to 13.62)	10.31 (8.99 to 11.83)	13.34 (11.61 to 15.32)	13.44 (11.74 to 15.4)	11.7 (10.21 to 13.4)	15.1 (13.19 to 17.29)

**2006**	11.24 (9.96 to 12.7)	9.78 (8.67 to 11.04)	12.63 (11.19 to 14.27)	8.65 (7.64 to 9.8)	7.51 (6.64 to 8.5)	9.73 (8.59 to 11.03)	9.32 (8.23 to 10.55)	8.09 (7.15 to 9.15)	10.49 (9.26 to 11.88)	11.93 (10.57 to 13.47)	10.37 (9.19 to 11.7)	13.42 (11.88 to 15.16)	13.43 (11.92 to 15.12)	11.69 (10.38 to 13.17)	15.08 (13.39 to 16.98)
**2007**	11.31 (10.15 to 12.6)	9.84 (8.84 to 10.95)	12.71 (11.4 to 14.16)	8.68 (7.77 to 9.7)	7.54 (6.75 to 8.42)	9.76 (8.73 to 10.92)	9.36 (8.38 to 10.46)	8.13 (7.28 to 9.07)	10.53 (9.42 to 11.78)	11.96 (10.73 to 13.32)	10.39 (9.33 to 11.57)	13.45 (12.06 to 14.99)	13.57 (12.22 to 15.07)	11.81 (10.64 to 13.12)	15.25 (13.73 to 16.93)
**2008**	11.37 (10.3 to 12.56)	9.89 (8.97 to 10.92)	12.78 (11.57 to 14.12)	8.71 (7.86 to 9.65)	7.56 (6.83 to 8.37)	9.8 (8.84 to 10.87)	9.4 (8.49 to 10.42)	8.17 (7.38 to 9.04)	10.58 (9.55 to 11.73)	11.98 (10.85 to 13.23)	10.41 (9.44 to 11.49)	13.47 (12.19 to 14.89)	13.72 (12.47 to 15.09)	11.94 (10.86 to 13.14)	15.42 (14.02 to 16.96)
**2009**	11.44 (10.4 to 12.59)	9.95 (9.05 to 10.94)	12.86 (11.68 to 14.16)	8.74 (7.91 to 9.66)	7.59 (6.88 to 8.38)	9.83 (8.89 to 10.87)	9.45 (8.56 to 10.43)	8.21 (7.44 to 9.05)	10.63 (9.62 to 11.75)	12 (10.91 to 13.22)	10.43 (9.49 to 11.47)	13.5 (12.26 to 14.88)	13.87 (12.66 to 15.21)	12.07 (11.01 to 13.23)	15.6 (14.23 to 17.1)
**2010**	11.51 (10.42 to 12.7)	10.01 (9.07 to 11.04)	12.93 (11.71 to 14.29)	8.77 (7.91 to 9.72)	7.62 (6.88 to 8.44)	9.87 (8.89 to 10.95)	9.49 (8.57 to 10.52)	8.24 (7.45 to 9.12)	10.68 (9.63 to 11.84)	12.03 (10.89 to 13.29)	10.45 (9.47 to 11.53)	13.53 (12.24 to 14.96)	14.03 (12.75 to 15.42)	12.21 (11.1 to 13.42)	15.77 (14.34 to 17.35)
**2011**	11.57 (10.39 to 12.9)	10.07 (9.04 to 11.21)	13.01 (11.67 to 14.5)	8.8 (7.87 to 9.85)	7.65 (6.85 to 8.55)	9.9 (8.84 to 11.08)	9.54 (8.53 to 10.66)	8.28 (7.42 to 9.25)	10.73 (9.59 to 12.01)	12.05 (10.81 to 13.44)	10.47 (9.41 to 11.66)	13.56 (12.16 to 15.13)	14.18 (12.77 to 15.74)	12.34 (11.12 to 13.7)	15.95 (14.37 to 17.71)
**2012**	11.64 (10.31 to 13.15)	10.13 (8.97 to 11.43)	13.09 (11.58 to 14.79)	8.83 (7.79 to 10.01)	7.68 (6.78 to 8.69)	9.93 (8.76 to 11.27)	9.58 (8.46 to 10.86)	8.32 (7.36 to 9.42)	10.78 (9.51 to 12.23)	12.08 (10.69 to 13.65)	10.49 (9.29 to 11.85)	13.59 (12.02 to 15.36)	14.33 (12.74 to 16.13)	12.47 (11.08 to 14.04)	16.13 (14.33 to 18.16)
**2013**	11.71 (10.19 to 13.45)	10.18 (8.88 to 11.69)	13.16 (11.46 to 15.13)	8.86 (7.69 to 10.21)	7.71 (6.7 to 8.87)	9.97 (8.65 to 11.5)	9.63 (8.36 to 11.09)	8.36 (7.27 to 9.63)	10.83 (9.4 to 12.49)	12.1 (10.53 to 13.9)	10.51 (9.16 to 12.07)	13.61 (11.84 to 15.65)	14.49 (12.66 to 16.59)	12.61 (11.02 to 14.43)	16.31 (14.25 to 18.68)
**2014**	11.77 (10.06 to 13.78)	10.24 (8.76 to 11.98)	13.24 (11.31 to 15.5)	8.89 (7.58 to 10.44)	7.73 (6.6 to 9.07)	10 (8.52 to 11.75)	9.67 (8.25 to 11.35)	8.4 (7.17 to 9.86)	10.88 (9.27 to 12.78)	12.12 (10.36 to 14.2)	10.53 (9 to 12.32)	13.64 (11.65 to 15.98)	14.65 (12.56 to 17.09)	12.75 (10.93 to 14.87)	16.49 (14.14 to 19.25)
**2015**	11.84 (9.92 to 14.14)	10.3 (8.64 to 12.3)	13.32 (11.15 to 15.91)	8.92 (7.45 to 10.69)	7.76 (6.49 to 9.29)	10.03 (8.38 to 12.03)	9.72 (8.12 to 11.64)	8.45 (7.06 to 10.11)	10.93 (9.13 to 13.1)	12.14 (10.17 to 14.51)	10.54 (8.84 to 12.59)	13.67 (11.44 to 16.34)	14.8 (12.44 to 17.63)	12.88 (10.83 to 15.34)	16.68 (14.01 to 19.86)
**2016**	11.91 (9.77 to 14.53)	10.36 (8.5 to 12.64)	13.39 (10.98 to 16.35)	8.95 (7.33 to 10.96)	7.79 (6.38 to 9.53)	10.07 (8.23 to 12.33)	9.76 (7.99 to 11.95)	8.49 (6.95 to 10.38)	10.98 (8.98 to 13.44)	12.17 (9.98 to 14.86)	10.56 (8.67 to 12.89)	13.69 (11.22 to 16.73)	14.96 (12.31 to 18.21)	13.02 (10.72 to 15.85)	16.86 (13.87 to 20.52)

Table S1. National childhood cancer ASRs by years of study and gender.

** Data in parenthesis are 95% Uncertainty Interval (UI)*
